# Microplastics in the Human Body: Exposure, Detection, and Risk of Carcinogenesis: A State-of-the-Art Review

**DOI:** 10.3390/cancers16213703

**Published:** 2024-11-01

**Authors:** Eliasz Dzierżyński, Piotr J. Gawlik, Damian Puźniak, Wojciech Flieger, Katarzyna Jóźwik, Grzegorz Teresiński, Alicja Forma, Paulina Wdowiak, Jacek Baj, Jolanta Flieger

**Affiliations:** 1 St. John’s Cancer Center, Department of Plastic Surgery, ul. Jaczewskiego 7, 20-090 Lublin, Poland; idziarzhynski@cozl.pl (E.D.);; 2 Institute of Health Sciences, John Paul II Catholic University of Lublin, Konstantynów 1 H, 20-708 Lublin, Poland; 3 Doctoral School, Medical University of Lublin, Aleje Racławickie 1, 20-059 Lublin, Poland; 4 Department of Neurosurgery and Paediatric Neurosurgery, ul. Jaczewskiego 8, 20-090 Lublin, Poland; 5 Department of Forensic Medicine, Medical University of Lublin, ul. Jaczewskiego 8b, 20-090 Lublin, Poland; grzegorzteresinski@umlub.pl (G.T.);; 6 Institute of Medical Sciences, John Paul the II Catholic University of Lublin, Konstantynów 1 H, 20-708 Lublin, Poland; paulina.wdowiak@kul.pl; 7 Department of Correct, Clinical and Imaging Anatomy, Medical University of Lublin, ul. Jaczewskiego 4, 20-090 Lublin, Poland; jacek.baj@umlub.pl; 8 Department of Analytical Chemistry, Medical University of Lublin, Chodźki 4a (Collegium Pharmaceuticum), 20-093 Lublin, Poland

**Keywords:** micro/nanoplastics, microplastic pollution, microplastic exposure, microplastic detection, microplastic toxicity, carcinogenesis

## Abstract

**Background:** Humans cannot avoid plastic exposure due to its ubiquitous presence in the natural environment. The waste generated is poorly biodegradable and exists in the form of MPs, which can enter the human body primarily through the digestive tract, respiratory tract, or damaged skin and accumulate in various tissues by crossing biological membrane barriers. There is an increasing amount of research on the health effects of MPs. Most literature reports focus on the impact of plastics on the respiratory, digestive, reproductive, hormonal, nervous, and immune systems, as well as the metabolic effects of MPs accumulation leading to epidemics of obesity, diabetes, hypertension, and non-alcoholic fatty liver disease. MPs, as xenobiotics, undergo ADMET processes in the body, i.e., absorption, distribution, metabolism, and excretion, which are not fully understood. Of particular concern are the carcinogenic chemicals added to plastics during manufacturing or adsorbed from the environment, such as chlorinated paraffins, phthalates, phenols, and bisphenols, which can be released when absorbed by the body. The continuous increase in NMP exposure has accelerated during the SARS-CoV-2 pandemic when there was a need to use single-use plastic products in daily life. Therefore, there is an urgent need to diagnose problems related to the health effects of MP exposure and detection. **Methods:** We collected eligible publications mainly from PubMed published between 2017 and 2024. **Results:** In this review, we summarize the current knowledge on potential sources and routes of exposure, translocation pathways, identification methods, and carcinogenic potential confirmed by in vitro and in vivo studies. Additionally, we discuss the limitations of studies such as contamination during sample preparation and instrumental limitations constraints affecting imaging quality and MPs detection sensitivity. **Conclusions:** The assessment of MP content in samples should be performed according to the appropriate procedure and analytical technique to ensure Quality and Control (QA/QC). It was confirmed that MPs can be absorbed and accumulated in distant tissues, leading to an inflammatory response and initiation of signaling pathways responsible for malignant transformation.

## Simple Summary

Environmental pollution caused by nano- and microplastics (MPs) is widespread and has become a global issue. There is a confirmed accumulation of MPs in animal and human tissues, raising concerns about potential health effects. The accumulation of NMPs in human tissues, as well as their genotoxicity, mutagenicity, and impact on cancer development, is a relatively new area of research that presents several challenges, mainly related to instrumental limitations and ensuring quality assurance and quality control (QA/QC) in studies of both exposure and subsequent fate in the body, such as translocation and possible accumulation.

## 1. Introduction

The term “microplastic” was used in 2004 by Thompson et al. [1] and refers to plastic particles ranging from 1 µm to 5 mm in diameter. Plastic particles can be categorized based on their size into nanoplastics, microplastics, mesoplastics, macroplastics, and megaplastics (Table 1) [1,2,3]. Plastics can be either thermosetting or thermoplastic, depending on their re-formability. Most of the world’s plastic production is thermoplastics, such as PE, PP, PVC, PS, and PET [4,5,6]. Approximately half of the output is PE, which comes in two types: low-density PE (LDPE) and high-density PE (HDPE) [7].

Plastics are in fact mixtures of polymers and many chemical compounds, so-called additives. Plastics are contaminated with substances that remain unremoved, i.e., residual monomers, by-products, and also those that adsorb onto the plastic surface from the environment, such as polycyclic aromatic hydrocarbons or phenol derivatives, etc. The number of additives that have been identified in the composition of plastics can be arranged from the highest of almost 1200 to the lowest of about 400 in the following order: PVC > PUR/PU > LDPE > thermoplastics > PP > HDPE > PET > PA > ABS > PS [8].

The European Environment Agency reports that in Western Europe, annual plastic consumption per person is almost 150 kg, three times more than the global average [9]. The high durability causes permanent contamination of ecosystems and the entire food chain with plastic, which degrades to smaller and smaller sizes [10]. 

The same agency states that yearly microplastic exposure per person ranges from 70,000 to over 120,000 particles [11]. The main route of exposure to MPs is air and drinking water, especially bottled water [12]. Lehel and Murphy in 2021 described the trophic transfer of microplastics and the potential threats to human health, including carcinogenicity, liver dysfunction, and endocrine disruption [13,14]. The toxic effects of MPs on the environment and human health are not fully understood. Many authors emphasize that microplastics release various chemicals used in their production, including chlorine, phthalates, bisphenols, and brominated flame retardants, but also absorb various pollutants, such as heavy metals and organic pollutants, becoming carriers of other environmental toxins [10,15,16,17,18,19]. Initial studies focused on plastic pollution in the aquatic environment. Many studies have been conducted on the determination of MPs in sewage sludge concerning the threat of land contamination [20]. Lofty et al. [21] in their work estimated soil contamination in Europe at 31,000–42,000 tons of plastic. The European Union recommends limited use of plastics, but there are no standards for food contamination with MPs. The Opinion of the European Food Safety Authority (EFSA) recommends systematic monitoring of micro/nanoplastics in food [22]. According to the report prepared based on the decade of research 2010–2020, the annual consumption of MPs by humans is about 1.42 × 10^5^–1.54 × 10^5^ particles (0.04 mm × 250 mm × 400 mm, density: 0.98 g/cm^3^) per person. The authors of the report, after recalculation, warn that this amount corresponds to the consumption of 50 plastic bags per person per year [23].

The MPs have been detected in the human body, e.g., placenta, lungs, liver, sputum, breast milk, feces, urine, and blood, and have prompted research to understand their health impact [24,25,26,27,28,29,30,31,32]. Reports published over the past 10 years have reported on some effects of human accumulation of MPs, including inflammation, oxidative stress, immunity suppression, promotion of carcinogenesis, and alteration of reproductive and cognitive functions [15,24,25,26]. 

Many reports focus on biomonitoring of different populations and speculating on potential health effects associated with environmental exposure to MP [17,33,34,35,36,37,38,39,40,41,42,43,44]. Due to the ubiquitous, unavoidable, and constant presence of MPs in the natural environment, workplace, and human diet, studies are still needed to assess the accumulation of MPs in human tissues and the health effects of this process.

The review aims to gather the most recent advancements in sample preparation and identification of MPs. It also discusses health risks, particularly the potential development of cancer due to MPs exposure. We have reviewed both in vivo and in vitro studies regarding the potential impact of MPs on carcinogenesis. The introduction describes the current data on the source of exposure, route of exposure, translocation of MPs in the body, and methods of analysis. A total of over a thousand manuscripts were evaluated, along with previously retrieved articles. Between 2017 and 2024, Hundreds of articles on the health effects of exposure to MPs were published. Among these, several dozen were review articles and meta-analyses, most of which are published in English and offering open access. After a thorough content analysis of the papers, only 16 included carcinogenesis caused by exposure to MPs [32,45,46,47,48,49,50,51,52,53,54,55,56,57,58,59]. Most of these reports broadly describe the health effects of exposure to MPs [32,45,46,51,52,53,55,56] or focus only on the mechanisms of carcinogenicity [48,50,57,58]. Individual reports collect data on the toxicity of selected microplastics, such as Zarus et al. [54], who in their review describe the liver carcinogenicity potential of PVC-MPs or highlight a specific mechanism induced by exposure to MPs, e.g., oxidative stress [49] as well as cell death and DNA damage [47]. Since, from the researcher’s point of view, a reliable evaluation of the results must be preceded by an unambiguous procedure and analytical technique, in our review, the collected evidence of the carcinogenicity of MPs was preceded by a description of the sample preparation and identification procedure based on the current state of knowledge. To standardize abbreviations, plastic particles ranging in size from nano- to micro- are designated with the common general abbreviation MPs.

## 2. Source of NMPs and Routes of Exposure

In 2019, a report on human exposure to MPs in the natural environment was published [60]. The report emphasized that plastic particles are widely distributed in air, soil, water, plants, and animals and are also present in the human diet. There have been several review articles on the routes and sources of human exposure to MPs [11,61]. The primary routes of exposure to MPs include ingestion of contaminated products, dermal contact, and inhalation [62]. The presence of MPs has been detected in many food products, drinking water and beverages, fruits, vegetables, salt, honey, sugar, and marine organisms such as fish and shellfish [63]. The process of trophic transport in the aquatic ecosystem from the lowest levels, including zooplanktonic organisms, to the higher levels of the food chain, including crustaceans, mollusks, and fish, results in the exposure of humans to MPs through the consumption of aquatic organisms. Van Cauwenberghe and Janssen in 2014 [64] calculated that up to 11,000 plastic particles can be ingested per year through the consumption of shellfish. However, this calculation was based on the fact that there were 0.42 plastic particles per gram of shellfish, taking into account the very high consumption of about 27 kg per year, which corresponds to a daily consumption of about 74 g of shellfish.

Plants consumed by humans are also exposed to contamination by MPs. Plant contamination occurs as a result of atmospheric precipitation, through contaminated water in the case of aquatic plants, or absorption through the rhizosphere in the case of soil plants. Model studies confirm the above possibilities of plant contamination with MPs. As shown by studies, nano- (<100 nm) and microplastics (0.2 µm) present in the soil matrix can penetrate the barriers of membranes and cell walls [19,34,65]. On the example of aquatic plants, i.e., duckweed (*Lemma minor*) and moss (*Sphagnum palustre* L.), it was shown that larger MPs with dimensions of 10–45 μm have increased adhesion to the surface of aquatic plants and are deposited, forming a polymer film [66]. Similar studies were conducted by Capozzi et al. [67] using MPs from polystyrene (PS) and the aquatic freshwater plant *S. palustre* L. Aquatic and terrestrial animals are exposed to MPs directly or through the food chain or trophic transfer [66,68].

Even if the food you eat is not contaminated with MPs, there is still a chance of being exposed to airborne microplastics during food preparation and consumption. According to studies by Cantarino et al. [69], this type of exposure could lead to ingesting 13,731 to 68,415 particles per year. Much attention has been paid to the study of the presence of MPs in drinking water and beverages. Review studies have summarized reports about MPs detected in water [70,71].

Seafood is the most commonly tested type of food for MPs, as it absorbs MPs by filtering seawater. A wide range of MPs in terms of density, type, shape, and size has been detected in the tissues of bivalves, shrimp, squid, and crabs [72,73]. Of great importance is the fact that these organisms are most often consumed whole, including the digestive tract, and therefore are a more dangerous vector of exposure to MPs for humans compared to large fish, from which fillets are mainly used for consumption [74]. However, a laboratory study confirmed the possibility of translocation of MPs from the digestive tract to fish tissues in the example of the European sea bass Dicentrarchus labrax [75]. Examples of food and beverage products in which MPs have been tested are summarized in Table 2.

It is possible to expose people to MPs by inhaling contaminated air. There are several reports of the presence of MPs in outdoor air but also indoors [34,111]. Measurements of air pollution from MPs began in 2016 [34]. For MPs to reach the respiratory system, they must be of a suitable size, i.e., longer than 5 μm, diameter less than 3 μm, and length-to-diameter ratio greater than 3:1 [112]. Vianello et al. used a thermal breathing mannequin (BTM) to estimate the number of particles that can enter the respiratory system within 24 h. It turns out, as the report states, that people absorb up to 272 particles from the air per day [113]. MPs detected in air samples present in the operating room [114]. Air was filtered onto 0.02 µm membranes and analyzed by Fourier-transform infrared spectroscopy (FTIR). The average number of MPs was 1924 ± 3105 MPs per square meter per day, with a range of 0–9258 MPs per square meter per day. Simultaneous measurements were taken in the anesthesia room, revealing an average of 541 ± 969 MPs m^−2^ day^−1^ with a range of 0–3368 MPs m^−2^ day^−1^. The most common polymer types included PE, PP, and nylon. Interestingly, no MPs were detected in the operating room outside working hours.

Another route of exposure to MPs is the so-called contact through penetration of skin pores (SP), sweat glands, hair follicles, or skin wounds [115]. In the case of human facial skin, pores have different sizes, i.e., 40–80 μm and about 5–10 μm in diameter, respectively [116], and are individual features. Translocation of MPs through human skin depends on their size, and according to some researchers, it is possible when the size is <4 nm [117,118]. Damaged skin allows the penetration of slightly larger MPs with a size of <45 nm [115,119,120]. In turn, Gautam et al. [121] argue that basically skin barrier crossing is possible for particles < 100 nm. Studies on pig skin have shown that negatively charged particles, even of a bigger size of 50 and 500 nm in size [119] and a mass of less than 500 daltons, are able to cross the skin barrier [120]. Dendritic cells (Langerhans cells) participate in the internalization of MPs by hair follicles [122]. However, this transport route covers a relatively small skin surface [115]. The penetration of the skin barrier, which is naturally protected by the stratum corneum of the epidermis, is increased by substances commonly used in cosmetics, such as urea, glycerol, and α-hydroxy acids [123]. A threat in this case is facial care products containing MP [124,125,126,127]. In body care cosmetics, plastics are added in the form of microspheres with a size of <2 mm. From a chemical point of view, these are PE, PP, and PS materials. [128]. Kaur’s 2018 study [126] tested wash gels, scrubs, face masks, and lotions manufactured in India. The study results confirm the presence of plastics mainly in the form of microbeads in 50% or more of the products. The study authors blame microbeads for causing color defects, creating tiny cracks in the skin that become gateways for bacteria. It is important to emphasize that MPs are intentionally added to various cosmetic products as viscosity regulators, emulsifiers, polishing agents, conditioning agents, exfoliants, abrasives, etc. [129]. Shahsavaripour et al. [130] studied the exposure of workers of plastic bag factories to MPs, including the skin of the hands and face. They collected samples from 19 people. The study showed that the number of particles identified in the collected samples increased almost twofold during the working day. In the case of workers wearing face masks, an increase of almost 10-fold was observed, which suggests that face masks are an additional source of contamination. 

Wu et al. [131] presented possible mechanisms of ocular surface damage by exposure to MPs. The review authors cite reports from animal studies using stereofluorescence microscopy imaging that showed the accumulation of MPs particles in the lower conjunctival sac and the presence of inflammatory cells and increased expression of inflammatory factors and cytokines (IL-1α, IL1-β, and IL-6) in a time-dependent manner. Studies of plastic in the human eye are sparse. An example is the study by Márquez García et al. [132] on MPs in contact lens waste and Zhong et al. [133], who found the presence of plastic in the vitreous humor of the eye collected from a cohort of 49 patients with different eye diseases (macular hole, macular epiretinal membrane, retinopathy, and rhegmatogenous retinal detachment). The study utilized laser-dimming infrared spectroscopy (LD-IR) and pyrolysis gas chromatography/mass spectrometry (Py-GC/MS). They identified 1745 plastic particles below 50 μm, mainly nylon 66, PVC, and PS. In addition, the report by Flieger et al. [134] found the presence of bisphenol A, used as a plasticizer during the production of plastics, in ocular fluid collected from patients undergoing cataract surgery.

## 3. MPs as Vectors of Toxic Substances

The cause of MPs toxicity is their translocation to various organs [135] and exposure to chemicals released from MPs [50,136,137]. In addition to the risk caused by the toxic effects of polymers and monomers and various additives released into the surrounding environment from MPs, plastics release chemical and microbiological contaminants that have been adhered to them due to their active huge surface, hydrophobicity, and surface functionalization [136,138,139]. The so-called “eco-corona”, originating from the environment, that covers MPs causes increased internalization [140] and an increased risk of attack by pathogens accumulated on their surface [141,142].

Plastics are mixtures of polymers and various additives necessary in the production process. The additives are chemical substances not covalently bound to the polymer so that they can be released over time [17]. Examples include bisphenol A (BPA), vinyl chloride, styrene, styrene-7,8-oxide (SO), triclosan, bisphenols, organotin, and phthalate esters used as plasticizers or brominated flame retardants (BFR) [143,144]. It should be emphasized that phthalate esters such as butyl benzyl phthalate (BBP) and di-2-ethylhexyl phthalate (DEHP) are considered carcinogens [4]. 

In addition to their toxicity, MPs are carriers of various toxic chemicals adsorbed on their surface from the environment due to their affinity for plastics, i.e., heavy metals and organic pollutants such as polycyclic aromatic hydrocarbons (PAHs), polychlorinated biphenyls (PCBs), and polybrominated diphenyl ethers (PBDEs) [17,18,19,145]. Both PCBs and PAHs may, among other adverse health effects, have genotoxic, mutagenic, and carcinogenic effects [146].

The interaction of MPs with various chemicals has been intensively studied. Verla et al. collected in a review possible interactions of MPs with different elements, i.e., Al, As, Cd, Cr, Co, Cu, Fe, Mn, Ni, Pb, Ti, Zn, Br, and organic pollutants with carcinogenic, teratogenic, and mutagenic effects, which are common in the anthropogenic environment, i.e., besides PAH, PCB, and PBDE, there are also dichlorodiphenyltrichloroethane (DDT), hexachlorocy-clohexane (HCH), chlordane, mirex, hexachlorobenzene, hopanes, perfluorinated compound/acid (PFC/PFA), aliphatic hydrocarbons, BPA, nonylphenols (NP), and octylphenols (OP) [17]. MPs adsorb per- and polyfluoroalkyl substances (PFAS) [147]. Hatinoglu et al.’s study [147] proved the adsorption of perfluorocarboxylic acids (PFCA) by polystyrene MPs (PS-MPs). The developed models based on linear solvation energy relationships (LSER) show that the main factors responsible for the adsorption of PFCA by MPs are the polarizability and hydrophobicity of the anionic PFCA. The presence of water and van der Waals interactions weaken the adsorption of acids by PS-MPs. It was observed that the presence of MPs in animal tissues is accompanied by persistent organic pollutants (POPs). An example can be the study of Herzke et al. [148], who examined tissues of northern fulmars (Fulmarus glacialis), which is recognized as an indicator species of MPs contamination by the Oslo-Paris Convention. In over 70% of tissue samples, liver, muscle tissue, and stomach, they detected PCBs, PBDEs, polybrominated biphenyl ethers and DDTs, dichlorodiphenyltrichloroethane, and various amounts of plastic particles from zero, medium (0.01–0.21 g), to high amounts (0.11–0.59 g). However, after analysis, they concluded that plastic is rarely a vector of POPs.

Another study is the study by Fossi et al. [149], who performed a skin biopsy of a whale shark (*Rhincodon typus*), which is an endangered species. They investigated the relationship between plastic debris in seawater samples in La Paz Bay, which ranged from 0.00 elements/m^3^ to 0.14 particles/m^3^, and substances associated by sorption, i.e., organochlorinated biphenyls (PCBs, DDT), and PBDEs. The concentrations of contaminants detected can be arranged in the following order: PCB > DDT > PBDE > HCB. The effect of contaminants on sharks was assessed using the biomarker CYP1A. Another study [150] assessed birds and fish in lakes in Switzerland for the content of plastics and additives and accompanying hydrophobic contaminants adsorbed onto MPs, as well as some potentially toxic additives. Different forms (fragments, pellets, cosmetic balls, ropes, fibers, foils, foams) and types (polypropylene, polyethylene, polystyrene, etc.) of MPs were identified in all examined samples.

Sharma et al. [151] investigated the health risk of exposure to MPs carrying toxic PAHs. The study investigated the ability of MPs to adsorb PAHs, which was determined to be from 46 to 236 μg/g within 45 min of contact time in aqueous solution. The authors of the study estimated the risk of cancer due to exposure to MPs to be higher than the recommended (10^−6^) and was 1.13 × 10^−5^ for children and 1.28 × 10^−5^ for adults. The study by Xiaojie Hu et al. [152] demonstrated that PE-MPs have the highest sorption capacity for carcinogenic PAHs, followed by PP and PS. After ingestion, these compounds are released in gastrointestinal fluids. The bioavailability of polycyclic aromatic hydrocarbons (PAHs) increases in the presence of mutagenic and carcinogenic phenanthrene and its derivatives, which are widespread in anthropogenic environments, making them highly bioaccessible. The authors reported that the lifetime risk of cancer following ingestion of PAH-contaminated MPs exceeds the safety limits set by the United States Environmental Protection Agency (USEPA).

Saraluck et al. [153] made an interesting observation while examining human milk samples. Comparison of the bacterial microbiota of human milk revealed taxonomic differences. The group in which MPs were detected had a more even distribution of bacteria, especially Bacteroides, compared to the group in which MPs were not detected. The authors of the study suggest that the detected bacteria might be linked to the detection of MPs.

## 4. Analytical Techniques for Detecting and Quantifying MPs

The presence of huge amounts of MPs in the environment means that humans cannot avoid contact with MPs and also with the products of their degradation into much smaller and more dangerous so-called secondary MPs. A crucial step in studying the impact of MPs on human health is to assess the presence of MPs in human biological samples, such as tissues and bodily fluids. There are increasing reports of MPs detection in various human samples [28,154,155]. 

Detection of MPs is an analytical challenge due to instrumental and methodological limitations. Detailed descriptions of the methodology can be found in review articles [68,156,157,158].

**Figure 1:**
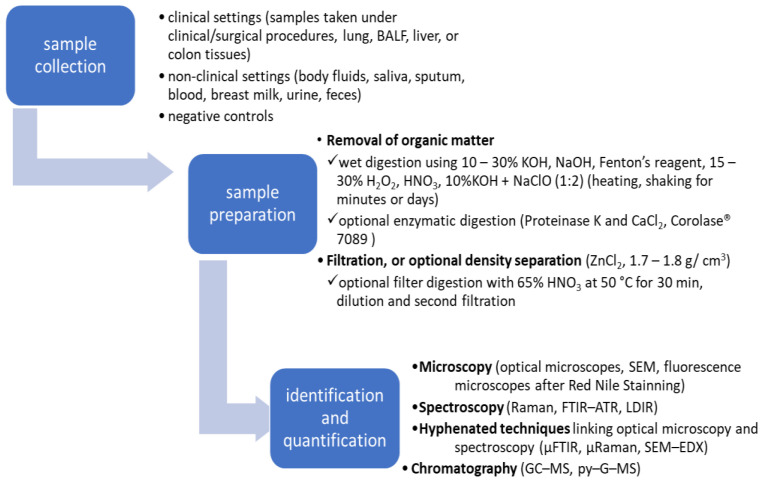
Scheme showing the subsequent steps of the analysis of MPs in human samples.

Studies conducted to date show that special care must be taken to avoid contamination during sampling, pre-treatment, and analysis. It is important to minimize sample cross-contamination due to the presence of MPs in the air as well as in the materials used during analysis. Sample contamination can be a source of false positive results. Reports of MPs determinations should include a description entitled: Quality Assurance and Control (QA/QC). The WHO report from 2022 [159] as well as other researchers [71,135,160,161] claim that most of the published studies do not meet the criteria (QA/QC), and the high uncertainty of the results makes them insufficient to estimate the risk of MPs to human health. Figure 1 illustrates the subsequent steps of the MPs analysis that will be discussed.

### 4.1. Sample Pretreatment

Preparation of biological samples, such as human samples, requires the removal of organic contaminants. Therefore, the first step is to wet digest the organic matrix. The digestion process can be carried out by wet oxidation using aggressive alkaline or acidic chemicals such as 10–30% potassium hydroxide (KOH), Fenton’s reagent (H_2_O_2_ + Fe^2+^), 65% nitric oxide (HNO_3_), hydrochloric acid (HCl), perchloric acid (HClO_4_), an acid blend of HNO_3_:HClO_4_ (4:1), oxidization with hydrogen peroxide (30% or 35% H_2_O_2_), and by enzymatic proteolysis using proteinase K and CaCl_2_ or enzymatic mixture Corolase^®^ 7089 [154,162]. However, it should be taken into account that acid and alkaline etching may lead to the destruction of MPs. Some strong acids can destroy polymers such as PS and PA, while alkaline etching destroys PA and PE fibers and may also lead to melting or discoloration of other polymers [156]. The digestion stage can last from several hours to several weeks and usually takes place at an elevated temperature of 40–65 °C. The next stage is density separation using high-density salt solutions as extraction media such as ZnCl_2_ (ρ = 1.6–1.7 g cm^−3^), NaI (ρ = 1.8 g cm^−3^), Na_6_[H_2_W_12_O_6_] (ρ = 1.4 g cm^−3^), and NaBr (ρ = 1.55 g cm^−3^) [148]. In many experiments, mixtures of reagents are used for digestion of organic matter and isolation of MPs, e.g., NaOH + HNO_3_ + Proteaze, 0.05% SDS + 5 mM CaCl_2_ + 1 M Tris HCl, 35% H_2_O_2_ + ZnCl_2_, 10% KOH + sodium hypochlorite, 30% H_2_O_2_ + 0.05 M NaOH, 30% H_2_O_2_ + 0.05 M Fenton reagent, 10% KOH + CHKO_2_ [163].

The next stage is centrifugation and filtration, usually using Al_2_O_3_ membranes, cellulose filters, silver membranes, glass fiber membranes, glass fiber filters, or even filter paper (Table 3). When using IR spectroscopy for identification in transmission or reflection mode, it is important to choose the right filter. Filters that transmit infrared are made of zinc selenide, aluminum oxide, polycarbonate, calcium fluoride, and barium fluoride. If the determination is performed in reflection mode, infrared refractive filters should be used, i.e., MirrIR Low-E slides, silver membranes, and gold-coated filters. Very often, LDIR is used for analyzing human tissue samples in transflective mode, requiring the use of infrared-reflective glass slides such as Kevley/MirrIR. The practice of transferring MPs to the appropriate filter is not recommended. Ourgaud et al. [164] recommend filtering through an IR-transparent filter and placing it on an IR-reflective filter to change the instrument mode.

Sample preparation requires avoiding contact with plastic materials. It is recommended to use cotton gloves, cotton towels, metal scissors, scalpels, and glass containers to reduce the possibility of sample contamination with plastic particles [154].

Good analytical practice is to perform a negative sample and screen all materials used in the experiment for MPs content and procedural blank. However, there is still a lack of optimized sampling and analysis procedures for MPs content, which results in an overestimation of their content in the tested samples. Many studies show that inadequate preventive measures are often implemented.

Jones et al. [191] conducted a study on the sources of MPs contamination in laboratory procedures. It turned out that the source of sample contamination can be water, airflow, and dust in the laboratory where significant amounts of MPs were detected, glass vessels, and also aluminum foil usually used to protect samples. Analysis by flow cytometry revealed that tap water contains more contaminants compared to the Milli-Q system and reverse osmosis. Surprisingly, the level of MPs contamination was lower when using plastic vessels instead of glass vessels (*p* < 0.0001). The difference is significant as the number of plastic particles detected in the experiment using glassware was 1356.9 (95% CI: 975.3–1861.1) particles/mL compared to the experiment using plasticware, which was 6.9 (95% CI: −0.7–19.2) particles/mL. The authors of the study evaluated the experiment performed on the laboratory bench. It turned out that the level of contamination reached a value of 55.6 (95% CI: 26–128.6) MPs particles/mL. Even the microbiological safety workbench did not prevent contamination of MPs samples. In laboratory dust samples, mainly PPs of sizes ~200 nm to > 10 µm were detected at a concentration of 1175 (96% CI: 884.5–1522.8) particles/mL. In comparison, much larger amounts of MPs were detected in homes. Soltani et al. [192] detected an average of 3095 plastic fibers/m^2^/ranging in size from 50 to 200 µm in Australian homes. The authors of the study suggest (i) performing procedural blind tests that allow for the examination of the number of MPs introduced during the experiment, (ii) minimizing the time of sample exposure to MPs, (iii) using plastic consumables, (iv) not using aluminum foil, (v) using Milli-Q water, (vi) performing experiments in a biological safety cabinet (BSC) or laminar flow cabinet (LAF bench), and (vii) removing laboratory dust using 70% ethanol and paper towel. The key element of detecting MPs in a sample is the choice of method. It is important to note the differences in the minimum detection size, e.g., for vibrational techniques, i.e., Raman or FTIR spectroscopy, the particle detection limit is ~1 μm and ~10 μm (for µ-FTIR it is about 2.7 µm), respectively, while flow cytometry is of the order of several hundred nm. Particles smaller than the detection limit will never be detected. The guidelines mentioned above are designed to ensure the quality (QA/QC) of studies on human samples involving MPs. Review articles have outlined the analytical challenges encountered at every stage of analyzing MPs in human samples [5,34,35,112,156,193,194,195,196,197,198]. The literature review reveals instances where authors have failed to utilize blank samples throughout the study or neglected to present results for control samples [199].

### 4.2. Physical Characterization (Visualization)

Visual inspection of MPs allows for determining the size, shape of particles, and the number of MPs with a size > 0.1 mm. Unfortunately, the identification of MPs with the naked eye is practically impossible. This method allows for the accurate identification of approximately 1.5% of visible particles. Usually, polymers are wrongly identified and misled with other particles, i.e., cellulose, ceramics, etc. [200,201]. 

The physical properties of MPs are primarily assessed using microscopic methods, including optical microscopy, stereoscopic and fluorescence microscopy after Red Nile staining of the samples, and polarized light microscopy. Stereo- or optical microscopes have a better resolution, which is less than 0.1 mm but greater than 1 µm.

In most of the works dedicated to the detection of plastics, transmission electron microscopy (TEM), scanning electron microscopy (SEM), and atomic force microscopy (AFM) are used [202,203].

AFM allows for imaging surface topography and evaluating cellular uptake and biodistribution of submicron plastic particles. In this instance, it is necessary to label plastic particles with fluorescent dyes. However, label-free techniques such as dark-field microscopy and reflected light hyperspectral microimaging are also viable options [204]. Nonetheless, there have been no reports of using AFM to image MPs in human cells thus far.

Scanning Electron Microscopy (SEM) allows for the examination of the shape of MPs and can also be utilized for identifying elements by incorporating an Energy Dispersive Spectroscopy (EDS) detector [205]. In X-ray SEM-EDS spectra, plastic particles exhibit a prominent carbon signal, making it possible to differentiate plastics from non-plastics [206].

### 4.3. Chemical Characterization

Chemical characterization of MPs can be achieved using vibrational spectroscopy techniques, such as Fourier-transform infrared spectroscopy (FTIR), Raman spectroscopy, or thermal techniques [203].

Thermal methods, i.e., pyrolysis gas chromatography coupled with mass spectrometry (py-GC–MS), thermogravimetry (TGA), TGA-MS, differential scanning calorimetry (DSC), TGA-thermal desorption TGA-GCMS (TGA-TD-GC–MS), TGA-DSC, thermal extraction desorption GCMS (TED-GCMS), and others provide analysis of MPs degradation products [207]. These techniques identify small-sized MPs in the unprocessed sample, destroying the sample and it is impossible to determine either the number or the shape and size of MPs [156]. And it is precisely the features of MPs such as size or shape, and the chemical nature of the surface that are crucial for toxicity [208]. 

FTIR and Raman spectroscopy [209,210] are commonly used to identify MPs, which have obvious instrumental limitations, mainly related to the size limits of detected particles and the possibility of generating false positive results [211,212,213]. Raman spectroscopy generates spectra that are the basis for identification similar to a fingerprint, providing information about MPs ranging from 0.5 µm to a few mm. Chemical composition is identified through characteristic bands or by comparison of the spectrum with reference spectra of polymers [214]. Raman spectroscopy enables the detection of particles smaller than 1 μm [215]. Interference from water is reduced because water has a low Raman signal, which is why a sample containing water can be analyzed [216]. The sample may not show strong fluorescence, which is possible in the case of colored and pigmented MPs; however, it is possible to remove background fluorescence using a special algorithm [217]. Improved resolution and sensitivity can be achieved by the hyperspectral imaging technique of stimulated Raman scattering (SRS) [218]. Raman microscopy (μ-Raman) combines light microscopy with Raman spectroscopy, allowing the study of particles > 0.5 μm. There is also the possibility of using a confocal Raman microscope offering spatial resolution in all three dimensions and characterization of samples in 3D [216].

FTIR is very often used to identify MPs with particle sizes above 20 µm based on the comparison of IR spectral bands with standard spectra in the library [201,203,219,220]. FTIR can work in three modes: transmission, reflection (i.e., transflectance and diffuse re-flection), and attenuated total reflectance (ATR), useful for determining MPs in aqueous solutions and biological samples that are placed on an ATR crystal [221,222]. Traditional FTIR, however, has a lower spatial resolution compared to Raman, which can lead to an underestimation of MPs content [221]. The detection range can be expanded using more advanced micro-FTIR (μ-FTIR) detecting particles < 10 µm and a focal plane array (FPA) detector. Focal plane array (FPA)-FTIR can detect and identify MPs < 20 µm [201,219,220]. Due to its high lateral resolution, (FPA)-FTIR generates multiple spectra simultaneously from the entire filter surface [223,224].

QCL-IR spectroscopy utilizes quantum cascade lasers (QCL) as a mid-IR radiation source to produce coherent radiation of specific wavelengths in the form of an intense and focused beam [225]. QCL in different versions covers different spectral ranges, such as a standard QCL (from 1800 to ~800 cm^−1^), a dual-range QCL (CH/FP) (from ~1800 to ~800 cm^−1^, and from −3000 to 2700 cm^−1^), and a QCL coupled to an optical parametric oscillator (OPO) (from 3600 to 2700 cm^−1^ and 1850 to 800 cm^−1^) [225]. Similar to FTIR, QCL-IR operates in transmission, reflection, and ATR modes. The advantage of QCL-IR is the possibility of selecting the IR range, which allows for quick identification. In QCL instruments, FPA can be used as a detector. The limitation of this approach is the risk of laser coherence, which generates various artifacts in the results, both images and spectra [226]. The most popular QCL-IR instrument used for NMPs analysis is Laser Direct Infrared (LDIR) (8700 LDIR, Agilent). It is used in environmental research to detect MPs [227,228] in food adulteration [229], food control [230], etc. The LDIR chemical imaging system measures particles in the size range of 20 to 500 µm.

Optical Photothermal Infrared Spectroscopy (O-PTIR) provides infrared spectroscopy and chemical imaging of microplastics from sub-microns to millimeters. This technique overcomes the limitations of conventional FTIR and Raman techniques, providing better chemical specificity and submicron spatial resolution. O-PTIR generates spectra independent of particle shape/size or sample roughness. O-PTIR spectroscopy acquires spectral information by inducing a photothermal effect using a QCL, which records a visible laser (532 nm or 785 nm) in the form of an O-PTIR spectrum. It is a “pump-probe” design. The quality of O-PTIR spectra is superior to FTIR spectra [231,232]. O-PTIR collects O-PTIR spectra, which are similar to FTIR spectra but is capable of collecting Raman spectra simultaneously, which makes identification more reliable [233]. Currently, there are various versions of O-PTIR microscopes available that provide qualitative and visual identification. Examples of O-PTIR instruments are the mIRage microscope and the mIRage+R microscope, both manufactured by Photothermal Spectroscopy Corp. Although O-PTIR offers the possibility to overcome the problems related to the interference of sample fluoresence, which distorts the spectrum in Raman analysis, or difficulties in identifying spectra due to the poor spatial resolution of FTIR, this technique is rarely used to detect MPs. An exception is the study by Su et al. [231], who used O-PTIR to identify MPs from silicone baby teats. Particles smaller than 600 nm were detected with a spatial resolution of about 400 nm.

Atomic force microscopy-based IR spectroscopy (AFM-IR), similar to advanced techniques with the exceptional resolution of about 20 nm, i.e., QCL-IR, O-PTIR, can analyze particles in the nano- to micro-size range [231,234,235,236]. Similar to O-PTIR, the mechanism of action is based on the photothermal effect, but the detection uses a detection probe in the form of an AFM cantilever [237]. AFM-IR can generate images for a single frequency, which allows the study of the surface structure of MNPs [237]. The AFM-IR instrument is the Nano IR2, which has a spectral range from ~3600 to ~900 cm^−1^.

In IR spectroscopy, hyperspectral imaging covering pixels in a selected area or collecting spectra only for localized MPs can be used. In MPs studies, FTIR spectroscopy equipped with an FPA detector is most frequently used, and AFM-IR is the least frequently used [225]. Correct identification of MPs is ensured by a properly selected spectral range and a sufficiently high spectral resolution, which depends on the instrument class and the researcher’s decision. Most researchers recommend a resolution of 8 cm^−1^ for studying MPs using FTIR [238]. When assessing the quality of spectra, attention should also be paid to the signal-to-noise ratio (SNR), which should be high, so that the signals are easier to identify. The SNR can be adjusted by the number of scans, laser power (IR), and the probe laser in O-PTIR. The problem of spectral quality is the so-called spectral artifacts distorting the spectra occurring in FTIR or QCL-IR spectra, e.g., the dispersion artifact, which occurs when the sample size is comparable to the wavelength, or artifacts occurring in the case of samples with rough surfaces [239,240]. Resonant scattering causing spectral artifacts does not occur in O-PTIR and AFM-IR techniques, which do not measure residual IR radiation.

Near-infrared (NIR) spectroscopy, due to its higher energy compared to IR, can penetrate to a greater depth. After absorbing radiation, MPs produce molecular overtones and combination vibrations. MPs are identified based on the resulting spectrum bands [203]. Near-infrared hyperspectral imaging (NIR-HSI) has been used to detect MPs with sizes larger than 300 to 150 μm [241] and up to 50 μm in environmental matrices [242,243]. Proton nuclear magnetic resonance (1H NMR) can also be used for the detection of MPs, but it is rarely utilized [244,245].

In the analysis of MPs in biological samples, it is necessary to determine the abundance, morphology (shape, size, surface character), and chemical identification of the polymer type. The combination of spectral data with spatial information is most useful for this purpose (Table 4).

## 5. Translocation of MNPs in the Animal/Human Body

Several reviews have been published where the results of studies have been collected, in which the presence of MPs in human tissue samples has been confirmed [45,135,154,163,246,247]. According to published reports, the dominant polymers found in the human samples are alkyd resin, nylon, EVA, CPE, rayon/viscose, resin, PA, PBS, PC, PE, PET, PMMA, PP, PS, PU, PVA, PVAc, and PVC, occurring as irregular fragments and fibers [154]. According to Roslan et al. [163], the size of particles found in human samples ranges from 10 to 4812.9 µm. Particles that are translocated, through cell membranes, to tissues are usually ≤10 µm in size.

Ingestion is the main route of human exposure to MPs. MPs contaminating food are excreted in feces [248]. The effect of MPs on the interaction between MPs and various biomolecules, microbiota [249], and lipid digestion in the gastrointestinal tract was studied in vitro in the gastrointestinal digestion model. The examinations included different plastic particles such as PS, PE, PVC, PET, and PLGA. The study results indicate that MPs affect the composition of microbiota and inhibit lipid digestion, with PS (50 nm, 1 µm, 10 µm) being the most active in that action [250]. In 2003, Liebmann et al. [251] detected the presence of PS, polyethylene glycol, and PU in stool samples obtained from participants who consumed a diet rich in seafood.

The bioavailability and processes of metabolism, translocation, and excretion of MPs are studied extensively. There are increasing reports indicating the systemic bioavailability of MPs in humans. Mohamed Nor et al. [252] estimate that a fraction of a percent, about 0.2–0.45% of consumed MPs, can cross the intestinal barrier. Moreover, only particles smaller than 150 µm in size can potentially penetrate the intestinal epithelium [22]. Larger MPs are retained in the intestinal mucus of the gastrointestinal tract. In a study involving volunteers, it was found that ingesting 15 g of PEMP plastic particles (sized 1–2 mm) led to an increase in gastrointestinal transit time [253]. This was attributed to the stimulation of enteric nerves and increased secretion from the upper gut. Stock et al. [254] conducted a study on the resistance of plastics such as PE, PP, PVC, PET, and PS to artificial digestive juices. The study suggested that these plastics exhibit high resistance to degradation in the digestive tract, indicating that they may be excreted completely in the feces without significant changes to their shapes and sizes.

MPs smaller than 2.5 mm are endocytosed in the gastrointestinal tract by the microfold cells of Peyer’s patches. The transfer of MPs to the systemic circulation is made easier in the case of pathological changes in the digestive tract, e.g., inflammatory diseases of the gastrointestinal mucosa, i.e., Crohn’s disease, peptic ulcer, etc. [255]. 

Systemic bioavailability and toxicity depend on the size of the plastic particles. Degradation leads to a decrease in particle size, increasing the surface-to-mass ratio and increasing both the reactivity and toxicity of MPs [256]. Studies on rats have confirmed that the smaller the particles, less than 100 nm, the greater the probability of systemic transport and reaching distant organs, such as the liver, spleen, lymph nodes, blood, and bone marrow [257]. In the case of particles larger than 10 µm, specialized phagocytes are required for transport, which translocates MPs by phagocytosis. Nonphagocytic cells are able to internalize particles < 1 µm in size. The mechanism of internalization is dependent on particle size and occurs via the clathrin- and caveolin-mediated endocytosis pathway. Surprisingly, studies on fish show that huge particles with sizes from 200 to 600 µm can also reach the liver [258].

The possibility of translocation of plastic particles regardless of the route of exposure is supported by animal studies. In a 2022 study by Sun et al. [259], male mice were given fluorescent PS beads of 100 nm and 3 µm in diameter by intravenous injection, gavage, or pulmonary perfusion. Urine samples were measured at 0.5, 1, 2, and 4 h after a single exposure by confocal laser scanning microscope (CLSM) and confirmed by transmission electron microscopy (TEM). Studies confirmed that MPs translocate to blood via the digestive and respiratory tracts and can be excreted in the urine. Pironti et al. [165] using µRaman analysis reported the presence of MPs in the urine of six individuals. They identified four MPs (sized 4–15 μm) as polyethylene vinyl acetate (PVA), PVC, PP, and PE. Some examples do not confirm the bioavailability and bioaccumulation of plastic particles in tissues or other measurable consequences of exposure to MPs. For example, while the results of Deng et al. [260] identified bioaccumulation of MPs in tissues after oral exposure to PS MPs (5 and 20 µm), Stock et al. [261] did not confirm such high oral bioavailability, as in mice most of the plastic was neither internalized nor translocated to distant organs and was excreted in the feces. In a mouse study, only a slight absorption of PS MPs (1, 4, and 10 µm) administered with food at a concentration of 10 mL/kg for 28 days by intestinal cells was confirmed. Rafiee et al. [262] did not observe neurobehavioral effects after oral exposure to PS (25 and 50 nM) at oral doses of 1, 3, 6, and 10 mg/kg/day for 35 days. In the study of Merski et al. [263], no bioaccumulation of PE and PET-MPs in tissues nor toxicity or mutagenicity were confirmed in rats fed 0.5%, 2.5%, and 5% MP for 13 weeks.

In contrast, there is ample evidence supporting the translocation of MPs into animal tissues in vivo studies [264]. An example is the study by Lu et al. [265], which involved exposing *Danio rerio* to PS and examining the accumulation of plastic particles in tissues. The authors confirmed that smaller PS particles of 5 µm in size accumulated in the intestines, gills, and liver, while larger particles of 20 µm were identified in the gills and intestines. Accumulation of plastic particles in liver tissues induced inflammation and accumulation of lipids and oxidative stress proteins. In turn, Peda et al. [266] used sea bass fed with 0.3 mm PVC for their studies. Three sections of the fish intestine were then subjected to histopathological examination after 1, 2, and 3 months. The inflammatory effect and structural changes caused by the movement of plastic particles through the muscularis mucosa and submucosa were confirmed.

MPs translocation across cell membranes depends on their shape and size. Particles of 20–20,000 nm can translocate from the gastrointestinal tract through the intestinal epithelial lining and from the trachea through the lung alveoli and then reach distant organs via the circulatory system [264,267]. Lee et al. [268] prepared a review on the absorption of MPs (<1 µm). The authors noted that most of the reports used spherical MPs with high concentrations and relatively short exposure times. The studies conducted showed that both absorption and health risks depend on the type, size, shape, concentration, and surface nature of the MPs. Health risks are greater in the case of exposure to smaller MPs with higher concentrations and sharp edges.

The movement of MPs into the bloodstream through the intestinal epithelium can occur through several mechanisms. These include endocytosis in the distal part of the intestine, transcytosis involving M cells of Peyer’s patches, and paracellular diffusion [7,188,269,270,271]. 

Exposure to MPs via the respiratory tract does not prevent translocation [36]. Inhaled particles, especially larger ones, can be mechanically excreted through both the gastrointestinal tract and the nostrils [272]. Alveolar macrophages play a role in the translocation of smaller MPs from the nostrils to the blood and lymph. The mechanisms involved in translocation include various types of endocytosis and diffusion [273,274]. After crossing the cell membrane barrier, MPs accumulate, initiating a state of oxidative stress and inflammatory reactions [275,276] or, after triggering defense mechanisms, are eliminated from the cell [264]. Due to their smaller size, MPs more easily penetrate membrane barriers due to transmucosal passage and are transported to distant tissues via blood and lymph [188,267].

There is evidence supporting the translocation of tissue membranes by MPs, as they have been found in various parts of the body, including the human placenta, tissues of the gastrointestinal tract, respiratory system, reproductive system, nervous system, blood, liver, kidneys, and spleen [188,264,277]. Published data indicates significant variability in polymer content across different tissues and organs in terms of type and size [278]. MPs > 50 µm were detected in the human placenta and meconium in a clinical setting by Braun et al. [279], providing clear evidence of translocation. Ragusa et al. [188] detected smaller MPs of 5 to 10 μm in most of the human placentas examined. Another report that confirms the translocation of plastic particles in human tissues is the 2023 study by Rotchell et al. [168]. The study was performed on saphenous vein tissues (n = 5) collected during surgery. Microplastic identification was performed using μFTIR spectroscopy (LOD 5 µm). The study meets the quality criteria on QA/QC in MPs analytics. The number of plastic particles detected in 4 of 5 vein tissue samples (14.99 ± 17.18 MPs/g) was reduced by the amount of particles detected in the empty samples (10.4 ± 9.21 MPs). Although there was no statistically significant difference between the empty and tested samples (*p* = 0.293), it was noted that the types of polymers were different in both types of samples. While irregularly shaped polymers such as alkyd resin (45%), polyvinyl propionate/acetate, PVAc (20%), and nylon-ethylene vinyl acetate, nylon-EVA, binder layer (20%) were dominant in the studied samples, mainly PTFE, PP, PET, and FNS were found in the controls. 

## 6. Cancerogenesis

MPs are highly toxic to cells due to their small size and high surface-to-volume ratio, which allows them to penetrate cells and interact with DNA and other macromolecules. MPs are internalized depending on the particle size, surface properties, and the so-called biomolecular corona responsible for the Trojan horse effect. Most authors pay special attention to the leakage of contaminants adhering to the MP surface and the hormezeutic effect, emphasizing the importance of the dose. Moreover, organic contaminants accumulating on the surface of plastics [280] and toxic metals used in production, such as As, Hg, Cd, Cr, and Pb, are equally responsible for carcinogenic effects. Most plastics (PS, PU, and PC) and additives (PVC, PCB, and PAH) are assigned to categories 1A and 1B, i.e., hazardous and carcinogenic substances [281].

In recent decades, it has been noticed that the number of both benign and malignant tumors in marine organisms, i.e., turtles, sea lions, and Tasmanian devils, is increasing [282,283], hence the authors’ suggestion to investigate the role of MPs contamination in inducing carcinogenesis in humans is understandable.

The cytotoxicity of MPs for the tested cells is unquestionable. The action of MPs disrupts cell homeostasis through many mechanisms, which include the induction of oxidative stress, damage to the biological membrane, activation of inflammatory factors, genotoxicity, and apoptosis [284]. For example, Prata et al. [24] or Chang et al. [285] indicate DNA damage and the involvement of inflammation induction involving the release of pro-inflammatory mediators after exposure to MPs.

MPs as environmental xenobiotics contribute to increased genome instability, leading to the initiation of new mechanisms and signaling pathways responsible for malignant transformation [137,286,287]. Most studies confirm DNA damage induced by MPs and interference with repair mechanisms, which clearly indicate the genotoxic potential of these particles [288,289,290,291]. It has been shown that chemicals that have been adsorbed on the surface of plastic particles, such as PAH contaminants and benzo[a]pyrene [291,292,293], play an important role in inducing DNA damage. Domenech et al. [46] in a review article analyzed data on the carcinogenicity of MPs. Most of the reports included in the review were in vitro experiments; in vivo studies were performed using rodent animal models. Most of these studies confirm the ability of MP to induce DNA damage, ROS generation, inflammation, genotoxicity, and metabolic disorders. They were performed in vitro on human cell lines [288,294,295] and in vivo on animal models [296,297], using mice, zebrafish, rats, etc. However, it should be emphasized that there are also studies that do not confirm DNA damage as a result of MP exposure [298,299]. Examples of studies from recent years are summarized in Table 5.

MPs induce DNA damage through different mechanisms (i) direct contact with DNA or indirectly through ROS generation, impairment of DNA replication, or impairment of DNA repair mechanisms. DNA damage and mutations can initiate the process of carcinogenesis [360]. The ability of MPs to damage DNA is confirmed by studies of the population of workers occupationally exposed to styrene. At the end of the 20th century, the occurrence of single-strand DNA breaks [361], the formation of micronuclei [362], chromosomal aberrations [363], etc. was reported in the studied group. However, there is still a lack of biomarkers to assess the carcinogenic activity of MPs in vivo and in vitro studies [364]. In 2020, Sharma et al. used the so-called toxicity equivalence factor to assess the risk of developing cancer after consumption of seafood contaminated with microplastics enriched with carcinogenic polycyclic aromatic hydrocarbons (PAHs) [151]. 

**Figure 2:**
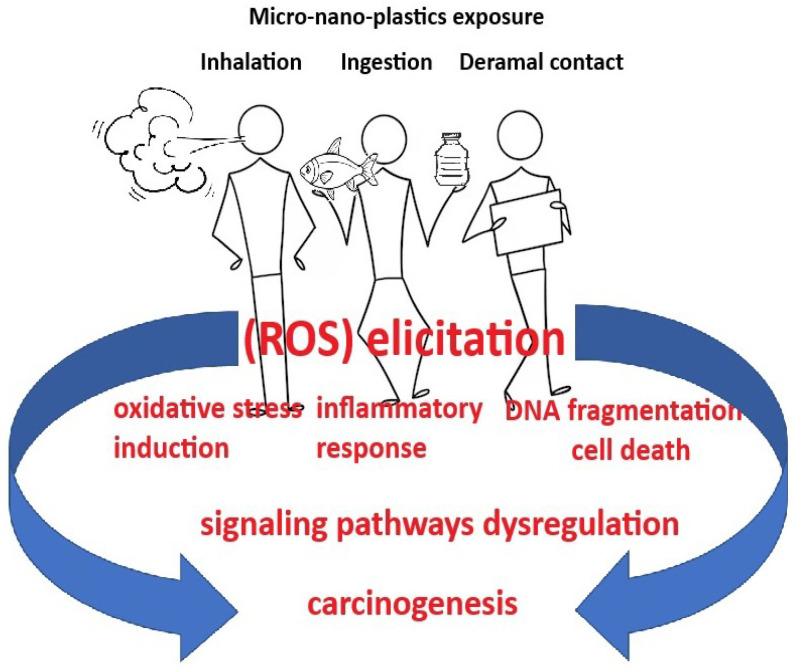
The health impacts of NMPs. The initiation of carcinogenesis is generated by abnormal gene expression and the influence of NMPs on various signaling pathways via cytokines (AP-1), interleukin-1 family: IL-1, IL-6, IL-8, tumor necrosis factor α (TNF-α), interferons (IFNs), Toll-like receptor 4 (TLR4), T helper 17 cell (Th17), suppressor T cells (Treg cells), interferon regulatory factors (IRF), activator protein 1 (AP-1) produced by inflammatory cells or via activation of intracellular kinases, i.e., the mammalian mitogen-activated protein kinase (MAPK) family: Extracellular signal-regulated kinases (ERKs), c-Jun N-terminal kinases (JNKs), and p38 mitogen-activated protein kinases (p38s), which dysregulate proliferation, differentiation, apoptosis, and stress response.

The effects of MPs range from DNA damage to inflammation [332] (Figure 2). The immune system recognizes MPs as foreign bodies, which are phagocytosed by macrophages and dendritic cells (DCs) [365]. Nienke Vrisekoop presented a study report [366] that showed that immune cells that come into contact with MPs die about three times faster than those that do not. This high mortality rate is higher than when immune cells are exposed to other foreign bodies or pathogens.

Alijagic et al. [367] showed that chronic exposure of human primary macrophages to polyamide-12 MPs increased the proinflammatory chemokine interleukin-8 (IL-8/CXCL-8). Based on the luciferase reporter gene study, it was determined that p53 was activated, indicative of a genotoxic stress state. Inflammatory cells create an oxidative environment by generating reactive oxygen species, i.e., O_2_^−•^, H_2_O_2_, and HO^•^ [368,369]. The association of inflammation with carcinogenesis occurs via multiple mechanisms, starting from genomic instability to pathological angiogenesis, tumor growth, and metastasis with the participation of angiogenic factors (vascular endothelial growth chemokines, NO, etc.) [370,371,372]. Therefore, cancers often arise at sites of infection that generate cytotoxic mediators. MPs cause local inflammation and secretion of various cytokines, including interleukin (IL)-1β, IL6, TNF-α, and IL-10 [373,374,375]. Such activity impairs, among other things, the host’s ability to fight pathogens [376,377]. 

To evaluate DNA damage by MPs exposure, indirect mechanisms related to genotoxicity are most often utilized, i.e., production of reactive oxygen and nitrogen species, induction of oxidative stress, alterations in cellular organelles, proteins, and genes, dysregulation in signal pathways [288,290,317,378,379]. 

Wu et al. [295] identified genes (Ras, ERK, MER, CDK4, cyclin D1, TRPV1, iNOS, IL-1β, IL-8) and 9NF-κB pathways, MAPK signaling, cytokine-cytokine receptor interaction, and Toll-like receptor) involved in proliferation and inflammation modulation, which are responsible for DNA damage and inflammatory diseases caused by exposure of the Caco-2 cell line to PS-MB. Figure 2 illustrates the initiation of carcinogenesis by NMPs.

**Figure 3:**
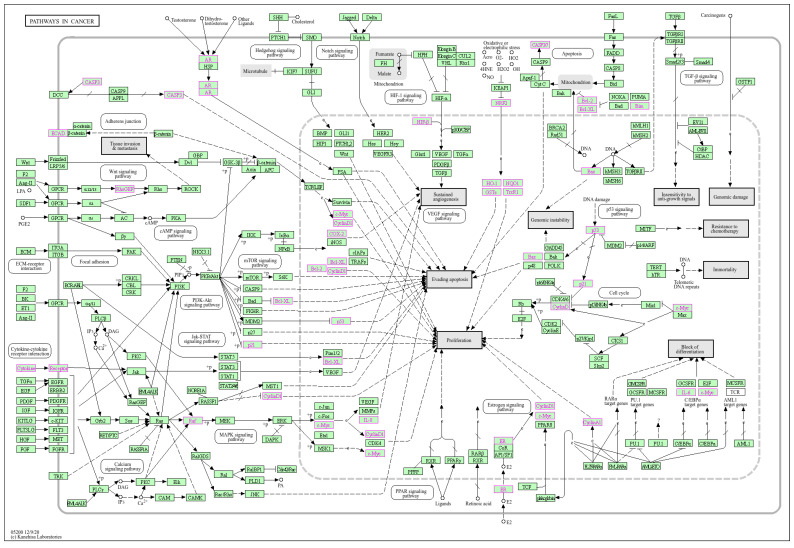
KEGG map of pathways in cancer (05200 Pathways in cancer) [380]. Rectangular boxes indicate gene enzymes. Genes marked in pink are identified to be up- or down-regulated by MPs additives [380].

Vincoff et al. [8] collected over 2700 additives and evaluated toxicogenomically the potential mechanisms of carcinogenicity and the influence of gene expression pathways. Genes that are up- and down-regulated as a result of exposure to MPs additives were selected. Up-regulated genes include the tumor suppressor TP53; pro-inflammatory cytokines (IL-8, IL-6); and regulators of detoxification, metabolism, and cell cycle (CYP1A1; CDKN1A). Genes with reduced expression are responsible for the regulation of apoptosis (BCL2, BCL2L1, BAX), cell adhesion, and an epithelial lineage marker (CDH1). Plastic additives share the same gene expression pathways as known carcinogens (Figure 3).

As can be seen, the effects of exposure to MPs additives include changes in the expression of genes involved in pro-inflammatory signaling pathways, oxidative stress, proliferation, apoptosis, induction of cell cycle arrest, and increasing the risk of carcinogenesis. The authors used the *k*-means method and hierarchical clustering of all additives and selected 3 clusters that similarly affect gene expression. The components of each cluster affect the cancer-like pathway (WP3859; WP4337), the immune pathway WP530 (cytokines and inflammatory response), the cell cycle/proliferation pathway (WP4357), the metabolic pathway (WP143, beta-oxidation of fatty acids), and the pathways regulating cell death/survival and DNA damage (WP3, WP3617, WP3617, WP3672) [8].

As seen in Figure 3, NMP Ps trigger multiple signaling cascades that are responsible for cellular damage and dysfunction of organs, lungs, heart, kidneys, liver, neurotoxicity, immunotoxicity, and reproductive toxicity. The changes concern signaling pathways that are marked in pink on the KEGG cancer pathway map, i.e., p53, MAPK, Nrf2, PI3K)/Akt, and TGF-β signaling pathways.

The first study devoted to the determination of MPs in human tumors (stomach, colon, lung, cervix, and pancreas) is the study by Zhao et al. from 2024 [381]. The authors identified three types of MPs in the examined tissues: PS, PVC, and PE using Py-GCMS. Among the 61 tumor samples collected, MPs were detected in 26 samples tested. MPs detection rates were 80%, 40%, 50%, and 17% (7.1–545.9 ng/g) in lung, stomach, colon, and cervical tumors, respectively. In pancreatic tumors, the rate was also high at 70% (18.4–427.1 ng/g). PS was detected in the largest number of samples, 20 samples (59.56 ± 89.15 ng/g), followed by PVC (51.98 ± 81.61 ng/g), which was identified in 17 samples, and the least common PE was detected in 11 samples (86.94 ± 116.84 ng/g). Lung cancer stood out in terms of high incidence, quantity, and types of detected MPs, while esophageal tumors did not detect MPs at all. The authors draw attention to the differences that occur in the tumor immune microenvironment (TIME) of pancreatic tumors without and with MPs. It was noted that in the pancreatic tumor samples where MPs were identified there were fewer anti-tumor cytotoxic cells, i.e., CD8+ T cells (*p* = 0.0023) and NK cells (NK; *p* = 0.0224), as well as a statistically significant reduction in dendritic cells (*p* = 0.0052) and an increase in the number of neutrophils (*p* = 0.0144). The report reveals different affinities of malignant tumors for MPs and that MPs affect TIME at least in pancreatic cancer. The changes observed in the tumor microenvironment may influence the efficacy of immunotherapy. Further studies are awaited in this direction. Moreover, MPs exposition has been linked with pancreatic, hepatic, biliary tract, and endocrine cancers [137,382].

### 6.1. Gastrointestinal Tract

The gastrointestinal tract is the main route of human exposure to MPs. Studies in simulated conditions have shown the potential for transfer of MPs through the food chain to higher trophic levels, increasing human exposure [383]. MPs enter the body through the consumption of contaminated food, beverages, and water [28,384]. 

The positive results of MPs determinations in feces are therefore understandable. Yan et al., confirmed the positive correlation between the amount of MPs particles in feces and the severity of inflammatory bowel disease (IBD) [184]. People with IBD have higher concentrations of MPs (41.8 items/g dm) in their feces compared to healthy individuals (28.0 items/g dm) [184]. The study authors report numerous microplastics (NMPs), with PET being the most common (22.3–34.0%) and PA following (8.9–12.4%).

Accumulation of MPs in ulcers of the rectal mucosa has also been reported [385]. Worrying data concern plastics industry workers, who have an increased risk of developing colon cancer [386] and a higher probability of death from pancreatic cancer [387].

In vivo studies in mice have confirmed that MPs and especially PVC cause disturbances in the composition of the microbiota [388], intestinal barrier dysfunction [389], and the development of inflammation [390]. This has its long-term consequences in the form of kidney, liver, and neurological disorders [391].

Studies involving mice have shown that MPs cause lysosomal damage and induction of IL-1β secretion by colonic macrophages. This creates an immune environment, the so-called tumor immune microenvironment (TIME), which consists of immune cells, cytokines, and extracellular matrix elements. In the TIME area, regulatory T cells and Th17 cells differentiate, which initiates and is responsible for the progression of colon cancer [392]. 

Growing evidence suggests intestinal toxicity and increased incidence of colorectal cancer (CRC) as a result of orally administered MPs [393]. Li et al. [394] suggest that MPs reduce the gut protective function by damaging the integrity of the colonic mucus.

Cetin et al. [395] studied the difference in the amount of MPs in colon tissue of patients diagnosed with colorectal adenocarcinoma compared to controls. The studies were performed using ATR-FTIR and Raman spectroscopy. MPs particles from 1 to 1299 μm were detected in colon tissues, which were identified as PE, PMMA, and nylon. In colon cancer tissue, there were 702.68 ± 504.26 particles/g, while non-cancerous tissues showed the presence of 207.78 ± 154.12 and 218.28 ± 213.05 particles/g. The significant difference detected between the content of MPs particles in cancer tissue compared to non-cancerous tissue indicates that exposure to MPs is associated with colon cancer.

A study conducted on colon cancer cells (HT29, HCT116, SW480, and SW620) [315] showed that PS-MPs accumulate in cells, can be transferred during cell division, and are not eliminated or degraded. Among the particles with different sizes of 0.25, 1, and 10 μm, the smallest MPs of 0.25 and 1 μm entered the cells and accumulated in lysosomes. Brynzak-Schreiber et al. [315] found that cells that internalized MPs became more mobile and increased migration. This finding may indicate that MPs may promote cancer metastasis.

Bruno et al. [393] prepared a review in which they gathered evidence confirming that ingested MPs may increase the risk of colorectal cancer (CRC), especially among patients with inflammatory bowel disease (IBD). The authors pointed out that MPs in the first stage cause damage to the intestinal epithelium and the development of intestinal inflammation. Certainly, the development of intestinal inflammation allows the internalization and translocation of MPs into the bloodstream, activating platelets, and then to distant organs.

Ibrahim et al. [181] reported the detection of MPs in colectomy specimens from 11 patients with colon cancer. MPs in the form of PC, PA, and PP filaments and fibers were detected in all samples at an average concentration of 28.1 ± 15.4 particles/g tissue.

MPs are also vectors that transport other carcinogenic toxins and genotoxic bacteria to the colonic epithelium, e.g., *Escherichia coli*, which increases the risk of colon cancer [396] after disruption of the internal mucus layer [397].

The studies of Bonanomi et al. [306] provide evidence for the carcinogenic potential of PS-MPs. The report shows that PS-MPs (2 μm in diameter, 20 μg/mL) and PS-MPs (0.5 μm in diameter, 5 μg/mL) are internalized by normal human intestinal CCD-18Co cells within 48 h but can be eliminated at the same time. In turn, chronic exposure for 4 weeks causes a persistent accumulation. The action of MPs is analogous to another carcinogen, azoxymethane (AOM), which causes metabolic changes by inducing oxidative stress and increasing glycolysis via lactate. 

There are few reports on the association of gastric cancer with MPs exposure. An exception is the report by Kim et al. [303] that linked chronic exposure to MPs with the risk of gastric cancer. The authors observed increased expression of the gene encoding the asialoglycoprotein receptor subunit ASGR2 (asialoglycoprotein receptor 2) after exposure of mice (NCI-N87) to PS-MPs at a concentration of 1.72 × 10^4^ particles/mL MP for 4 weeks. The results showed that even a single dose remained in the stomach for 24 h. Four-week exposure caused 2.9-fold faster migration of NCI-N87 cells. E-cadherin and N-cadherin expression, N-cadherin, and CD44 were also increased, which caused resistance to drugs such as bortezomib, paclitaxel, gefitinib, lapatinib, and trastuzumab.

Bisphenol A, added as a plasticizer in the production of plastics, when absorbed orally, appears to cause local inflammation and weaken the intestinal barrier function [398]. Mice exposed to bisphenol A (50 μg/kg body weight/day) showed reduced expression of lysozyme in the ileum. Perinatal exposure of pregnant mice increased colonic permeability in the offspring by increasing the level of, among others, interleukin-17, which is responsible for the resistance of cancer cells to drugs [399]. The pro-tumor inflammation in the human colon induced by bisphenol A exposure was a result of its binding to estrogen receptor beta, impairing the activation of the apoptotic cascade [400].

### 6.2. Respiratory System

Already at the end of the 20th century, the presence of fibers of unknown origin was detected in non-pathological and cancerous lung tissue [401]. In a 2021 study, MPs were detected in the form of fragments (3.9 ± 0.7 μm) and fibers (11 ± 2 μm) in 13 of 20 samples using Raman spectroscopy in post-mortem lung tissues [402]. Despite the fact that according to Cooper and Loxham [403] particles larger than 10 µm should be retained in the nasopharynx, such particles were detected in lung tissue (mean particle length: 105.22 ± 92.82 μm, mean particle width: 34.44 ± 22.61 μm) [178]. In sputum, particles of identified polymers were <500 μm (median: 75.43 μm) [190]. 

Studies by many research groups have shown that the accumulation of MPs in the lungs induces morphological changes and disrupts cell proliferation [177,319]. Many publications describe the development of respiratory diseases caused by exposure to plastics. This applies especially to occupational exposure to styrene, which exceeds 20 ppm. Already at the end of the 20th century, a retrospective study was conducted on over 3000 workers with an average age of 44 years occupationally exposed to styrene. During only one year of observation in this group, over 500 cases of invasive cancer were recorded, an increase in cases of tracheal, bronchial, and lung cancer. The study has many limitations in the form of lack of information, e.g., on lifestyle, smoking, etc. [404]. Similar results were described in studies from Great Britain [405], but many others do not confirm the association of styrene exposure with the development of lung cancer [406,407]. Studies in mice exposed to 160 ppm of styrene confirmed the carcinogenic potential of styrene [408].

An example confirming the association between exposure to and an increase in lung cancer incidence is a case-control cohort study conducted among 1658 workers exposed to PVC dust and/or vinyl chloride monomer (VCM) from 2003 [409]. The risk of lung cancer in workers exposed to PVC was assessed using the odds ratio (OR) in a logistic regression model. It turned out that the risk of the disease increased by 20% with each additional year of work in a workplace with exposure to PVC dust (OR = 1.2003; 95% CI 1.0772 to 1.3469; *p* = 0.0010). Such an association was not found for VCM exposure.

In 2017, Nett et al. [410] reviewed studies confirming that styrene exposure may be a potential risk factor for nonmalignant respiratory disease (NMRD). Examples of case reports include the diagnosis of an interstitial lung disease called occupational hypersensitivity pneumonitis (OHP) due to exposure to DMP and styrene [411], or terephthalic acid and dimethyl terephthalate (DMP), a precursor in the production of PET [412]. There are case reports of obstructive bronchiolitis in workers involved in the production of glass fibers using styrenic resins [413]. Respiratory problems and deterioration of pulmonary function tests, increased serum levels of interleukin (IL)-8 and tumor necrosis factor alpha (TNF-α), peribronchial thickening, and diffuse ground glass attenuation have been reported in association with PP exposure among workers in Turkey [414]. Inhalation of polyacrylic NPs has caused dyspnea and pleural effusion [415]. In turn, exposure to nylon fibers correlated with the severity and progression of interstitial lung disease based on a ten-year follow-up of workers in a flocking plant [416]. As reported by House et al. [417], the risk of bronchial asthma may occur due to exposure to thermoplastic acrylonitrile-butadiene-styrene fibers used for 3D printing.

Valavanidis et al. [418], in their review, gathered evidence for the leading role of oxidative stress and overproduction of reactive oxygen or nitrogen species (ROS, RNS) in the production of inflammatory mediators (MAPK family, NF-κB, AP-1) in the lungs and the initiation of carcinogenesis as a result of exposure to inhaled particulate matter of aerodynamic diameter 10 and 2.5 μm (PM 10 and PM 2.5).

In vitro studies have shown that PM causes DNA damage in lung epithelial cells [419,420]. The DNA damage manifested as strand breaks and formation of 8-hydroxy-2′-deoxyguanosine (8-OHdG).

In the study by Chen et al. [179], it was proven that MPs can contribute to the formation of ground-glass nodules (GGN). GGN is detected in preinvasive lesions, e.g., atypical adenomatous hyperplasia (AAH), adenocarcinoma in situ (AIS), minimally invasive adenocarcinoma (MIA), and lepidic-predominant invasive adenocarcinomas (LPA). In human GGN tissue samples, 65 microfibers, including 24 MPs (>20 μm), were detected by μ-FTIR. In control tissue samples, polyester and viscose were detected, while other types of MPs, such as acrylic, polyethylene glycol terephthalate (PET), and phenoxy resin, appeared in GGN samples. The authors suggest a possible association of GGN with MPs accumulation at the probability level of *p* = 0.0882.

### 6.3. Blood Neoplasms

In Denmark, data from approximately 73,000 workers associated with the plastics industry were analyzed. The study aimed to assess the risk of lymphohematopoietic malignancies associated with styrene exposure [421]. It turned out that the risk of acute myeloid leukemia, but not Hodgkin’s lymphoma or T-cell lymphoma, doubled in people exposed to styrene during the previous 15–29 years.

Similar data from American boatbuilders occupationally exposed to styrene was analyzed by Daniels and Bertke in 2020 [422]. An association was found between styrene exposure and the occurrence of leukemia and bladder cancer. Exposure to a fairly low level of styrene, namely 0.05 ppm, resulted in an additional death per 10,000 workers from hematological cancer.

The key factor contributing to the increase in cancer incidence is the duration and intensity of styrene exposure. This conclusion was made by Bertke et al. [423] based on a 2018 cohort study of over 5000 workers occupationally exposed to styrene.

Salvia et al. [424] measured NPs accumulation in human peripheral blood using flow cytometry based on fluorescent staining with a red phenoxazone dye called Nile red (NR). The presence of plastics such as low-density PE, PS, PET, and polyamide was examined in the blood of healthy donors (n = 37), neonates (n = 36), patients with multiple myeloma (n = 28), acute myeloid leukemia (n = 46), acute lymphoblastic leukemia (n = 26), chronic lymphoblastic leukemia (n = 16), non-small cell lung cancer (n = 16), idiopathic nephrotic syndrome (n = 9), and type 1 diabetes (n = 10). The results obtained in the study indicate a fairly large scatter of results. In healthy donors, with an average value of m = 667 events/µL, the scatter was 88–1460 events/µL. Although the authors declare that the highest MPs levels were found in patients with acute lymphoblastic leukemia (m = 648.3, r = 188–1354 events/µL), there was no statistically significant difference between the studied groups.

There is no doubt that the detection of MPs in the human bloodstream [176] indicates the risk of hematotoxicity [38]. Sun et al. [425] in an in vivo study in mice showed that exposure to (0.1 mg and 0.5 mg) of 5 µm PS-MPs caused a decrease in the number of white blood cells in peripheral blood and a decreased ability of colony-forming units CFU-G, CFU-M, and CFU-GM to form colonies in bone marrow cells, increasing the number of Pit. Increasing the dose from 0.1 to 0.5 mg resulted in a change of 41 to 32 differentially expressed genes (DEGs), respectively. Altered gene expression causes disruption of relevant metabolic pathways (Jak/Stat, pentose and glucuronide interconversions, nicotinate and nicotinamide metabolism, unsaturated fatty acid biosynthesis and the pentose phosphate pathway, T-cell homeostasis.72), leading to hematotoxicity.

### 6.4. Liver Carcinogenicity/Biliary Tract Cancer

In 2022, Im et al. [426] visualized the PS absorption pathway using PET imaging and demonstrated the accumulation of MPs in the liver of mice 24 h after orally administering 64Cu-Labeled PS. The effect of liver exposure to PS-MPs, sized 5 μm and 0.5 μm, was studied in a mouse model by Zou et al. [427]. TEM studies after hematoxylin and eosin staining showed structural changes in liver tissue. Among others, nuclear wrinkling and mitochondrial vacuolization were observed, which depended on the size of MPs and were more severe for larger MPs. The state of oxidative stress in hepatocytes was confirmed based on the decrease in the expression of sirtuin 3 (SIRT3) and superoxide dismutase (SOD2) proteins. Roh et al. [359] investigated the effect of 9-week exposure to 500 nm PS-MPs on liver metabolic function in an in vivo mouse model. Changes in lipid regulatory pathways (accumulation, adipogenesis, lipogenesis, and lipolysis), the disruption of amino acids, hepatic Glu metabolism, and insulin resistance were all detected in mice treated with PS-MPs. Increased leptin levels and the GLUT4-AMPK signaling pathway were observed. The effect of PS-MPs treatment on liver metabolism was verified in the HepG2 cells and MDI-stimulated 3T3-L1 adipocytes.

The authors of the reports suggest the accumulation of MPs in the liver as an organ where detoxification of various substances takes place and the adverse effect of MPs on liver metabolism. The carcinogenic effect, especially of substances released from MPs, was also confirmed. Vinyl chloride is used in the production of PVC, which is a carcinogenic substance. There are reports confirming the influence of vinyl chloride on the development of rare liver cancers such as hepatic angiosarcoma and hepatocellular carcinoma [428,429]. Zarus et al. [54] conducted an analysis of 34 reports documenting the effects of workers’ exposure to microplastics, especially vinyl chloride, used in the production of PVC, in the workplace. Comparison of results indicates a carcinogenic potential of MPs for the liver (angiosarcomas, hepatocellular carcinomas, and other neoplasms). A similar conclusion was reached in the report of the Agency for Toxic Substances and Disease Registry (ATSDR) prepared by the U.S. Department of Health and Human Services (HHS) [430]. The above studies indicate a long latency period, 27–47 years, for this type of cancer caused by exposure to vinyl chloride. The effects of exposure to vinyl chloride and PVC are, however, much wider and also involve the respiratory system. Various types of lung cancer (squamous cell carcinoma and adenocarcinoma) have been recorded among people exposed to high levels of PVC-MPs [409]. In 2010, the U.S. Department of Health and Human Services published a toxicological profile of styrene and PS [431]. The report included the effects of styrene on the immune system, lymphoreticular, neurological effects, reproductive effects, and cancer. Regarding the liver, the authors point out inconsistent results regarding the hepatotoxicity of styrene. In a study of animals exposed to 400 mg/kg styrene for 100 days, small areas of focal necrosis were observed in the liver [432]. Liver dysfunction was assessed based on the level of enzymes in the blood.

Ahrens et al. [433] examined the association between the motor, extrahepatic, and executive pathways for 14 endocrine disrupting effects. The study was conducted in 1995–1997 in six countries (Denmark, France, Germany, Italy, Spain, and Sweden) and included 183 cases and 1938 controls. The results of an unconditional regression test suggested that PCB exposure was responsible for the risk of extrahepatic bile duct cancer and ampulla of Vater. Odds ratios (OR) were plotted at 2.8 and 95% confidence intervals (95% CI 1.3–5.9).

Rosellini et al. [434] demonstrated the hepatotoxic effects of MPs on liver cells overexpressing cytochrome P450 monooxygenases (CYPs), an enzyme involved in the metabolism of xenobiotics. Molecular docking of over 1000 compounds identified as 2,2′-methylenebis(6-tert-butyl-4-methylphenol), 1,1-bis(3,5-di-tert-butyl-2-hydroxyphenyl)ethane, and 2,2′-methylenebis(6-cyclohexyl-4-methylphenol) that interact with CYP3A4. The isolated plastic-bound chemicals in vitro upregulated CYP3A4 gene expression, suppressed mitotic and “DNA-based DNA replication”, and rmetabolic and inflammation-related pathways. Another report described the exposure of human liver-derived pluripotent stem (LO) cells to 1 μm PS-MPs microspheres [329]. The genes responsible for MPs hepatotoxicity were identified, namely *HNF4A* and *CYP2E1*, which are upregulated following exposure.

### 6.5. Bladder Cancer

According to the World Cancer Research Fund International [435], bladder cancer is the 9th most common cancer worldwide. It affects men more often than women. In 2022, more than half a million new cases of bladder cancer were reported. Krafft, et al. [436] conducted a study of samples taken from 10 patients during bladder resection. Cancer (n = 10) and control (n = 10) samples were subjected to Raman microspectroscopy analysis with hyper-spectral imaging performed in the range of 600–1800 cm^−1^. In more than half of the samples (13 out of 20 samples), PS-MPs were detected. This is the first detection of MPs in bladder cancer tissue samples. The Know-it-all spectral library was used to identify the spectra, but no information was provided on the morphology of microplastic particles. The authors proposed several possible interpretations of the results, indicating the possibility of accumulation of plastic particles in the bladder, which may be one of the factors in the development of cancer, which in routine tests, i.e., histopathology, immunohistochemistry, and fluorescence, MPs were not taken into account. One of them explains the presence of MPs as a consequence of consumption of contaminated food and the possibility of excreting some of the absorbed MPs in urine. Another explanation, which the authors allow, is the contamination of samples during collection, handling, and analysis, especially in the case of PS and cellulose fibers. An obvious limitation of the study is the detection limit of the Raman spectroscopy used, which prevents detection of particles smaller than hundreds of micrometers, which causes the reported MPs values to be underestimated.

### 6.6. Skin Cancer

Environmental toxins play an important role in the etiology of squamous cell carcinoma (CSCC) [437]. Wang et al. [314] in an in vitro study using squamous cell carcinoma cell lines (SCL-1 and A431) determined the effect of MPs on skin cancer. In the study, PE-MPs (1 µm) was used at a concentration of 0–1 mg/mL. This concentration was selected based on the previous study by Wang et al. [438], who determined the average environmental exposure to MPs. It was shown that the MPs particles used were internalized into squamous cell carcinoma cells. Internalization was dependent on the exposure time and exposure dose. The highest value of fluorescence was measured after 60 min of contact time. In the study of the effect of MPs on skin cancer cells, a number of methods were used, i.e., MTT, flow cytometry, confocal laser microscopy, Western blotting, and others. MTT analysis confirmed that MPs promote skin cancer cell proliferation. In a study using flow cytometry, an increased cell cycle progression (decrease in the number of cells in the G1 phase, increase in the percentage of cells in the S and G2 phases) was demonstrated as a result of MPs exposure. An increase in the expression of CyclinD1, c-Myc, and Bcl-2 proteins associated with the cell cycle and a marker of cell proliferation (Ki67) was also observed. Studies of the mechanism by which MPs affect skin cancer cell proliferation confirmed the participation of the inflammatory response due to the activation of the NLRP3 inflammasome under the influence of mtDNA and an increase in TNFα, IL-6, and IL-1β. The initiating role in this process is played by excessive ROS production in mitochondria and a change in the mitochondrial membrane potential under the influence of MPs. The authors do not exclude the involvement of other inflammatory signaling pathways that are activated by MPs and are reported by other authors, such as NF-κB or cGAS-STING [439,440,441].

In the study by Wang et al. [314], the damaging effect of MPs on normal skin cells (HaCaT) was also assessed. It was found that MPs increased the expression of inflammatory factors, but in contrast to squamous cell carcinoma cells, the proliferation of HaCaT cells was inhibited. The authors presented an interesting interpretation of this fact. The authors believe that the damage to normal cells is associated with cell pyroptosis through the activation of GSDMD. While MPs stimulation in normal cells induces pyroptosis mediated by GSDMD, in skin cancer cells pyroptosis does not occur because the expression of GSDMD is too low. Further studies are required to confirm that MPs are capable of damaging the skin and promoting the proliferation of cancer cells.

### 6.7. Breast Cancer

MPs are rich in endocrine disrupting substances (EDCs). In the study by Park et al. [301], the effect of PP-MPs in the size of 16.4 µm and concentration of 1.6 mg/mL was investigated on human breast cancer cell lines, MDA-MB-231 and MCF-7. The authors did not observe the effect of MPs on cell mortability and motility. However, prolonged incubation (12 and 24 h) of PPMPs and MDA-MB-231 and MCF-7 accelerated the cell cycle and increased the expression of TMBIM6, AP2M1, and PTP4A2 genes and interleukin 6 (IL-6), while the level of the FTH1 transcript decreased in breast cancer cells. The authors warn that there is a possibility of tumor progression and metastasis as a result of chronic exposure to PPMPs.

Böckers et al. [442] investigated the endocrine potential of one of the most commonly used plasticizers, the organophosphate ester tri-o-cresyl phosphate (TOCP). The in vitro study used HEK-ESR1 cells transfected with estrogen receptor α (ERα) and the human breast cancer cell line MCF-7. TOCP was identified as an ERα ligand comparable to 17-β-estradiol. ESR1-related genes with altered expression were identified that increase angiogenesis and promote tumor growth and metastasis.

The carcinogenic effects of bisphenol A and tricresyl phosphate were confirmed by Deng et al. [443] in a human breast cancer cell line (MCF-7). Studies have revealed that exposure to bisphenol A in breast cancer cells causes increased proliferation due to decreased expression of miR381-3p and increased expression of pituitary tumor transforming gene 1 (PTTG1) protein due to inhibition of microRNA (miR-381-3p). Exposure to bisphenol A causes double-stranded DNA breakage, causing genomic instability and the risk of cancer development [444].

### 6.8. Kidney Cancer

Karami et al. [445] showed an association between exposure to polycyclic aromatic hydrocarbons as well as styrene and acrylonitrile and an increased risk of renal cell carcinoma. Similar conclusions were presented by Dahman et al. [446] based on a meta-analysis of reports on the risk of kidney cancer and exposure to increased levels of PM 10 air pollution. La Porta et al. [278] in a comprehensive review collected studies on the risk of kidney damage and exposure to MPs. The authors gathered studies in animal models that supported the possibility of renal dysfunction and damage resulting from MPs exposure caused by the induction of inflammation, oxidative stress, autophagy, apoptosis, and fibrosis. An example is the report by Zou et al. [447], which describes kidney damage as the effect of exposure of mice to 10 mg/L of 5 μm MPs and 50 mg/L of CdCl_2_ for 3 months on kidney function. 

Javeria Zaheer et al. [448] used PET imaging to identify renal dysfunction in a mouse model exposed to PE at a dose of 0.1 mg/mL/100 μL for a period of 12 weeks. The authors showed that PE accumulated in the kidney and increased the expression of Myc, CD44, programmed death ligand 1 (PD-L1), and hypoxia-inducible factor (HIF)-1α. In addition, the occurrence of renal failure and increased glucose metabolism were confirmed. The observed changes may result in the development of cancer in the future.

The next study is the study by Xu et al. [449] from 2024. In a mouse model, the authors observed the effects of PS-MPs exposure in animals with a high-fat diet (HFD) for 30 days on kidney development. Based on single-cell RNA sequencing (scRNA-seq), they showed kidney damage. PS-MPs plus HFD treatment by generating ROS and inflammation changed the organization of proximal and distal convoluted tubule cells and induced carcinogenesis. The profibrotic and protumorigenic regulation of the microenvironment was mediated by PF4 + macrophages. Furthermore, PS-MPs plus HFD induced activated PI3K-Akt, MAPK, and IL-17 signaling pathways in endothelial cells and increased the proportions of effector CD8 + T cells and proliferating T cells.

### 6.9. Brain Tumors

The blood-brain barrier (BBB) essentially prevents MPs and other substances from the blood from entering the extracellular fluid of the central nervous system. However, animal studies have shown that MPs are able to reach the brain as a result of disruption of the BBB after oral administration or injection [450,451]. The effects of accumulation have been microglial activation, neuronal damage, impaired cognitive functions, anxiety, and depressive disorders [451,452,453]. Accumulation of PS-MPs has been confirmed to cause the progression of Parkinson’s and Alzheimer’s diseases [454] by promoting aggregation of α-synuclein in dopaminergic neurons. In the study by Gaspar et al. [455], the accumulation of MPs in the brains of mice was confirmed after oral administration. Mice were exposed for 24, 48, and 72 h to 0.1 and 2 μm PS-MPs at concentrations ranging from 0.01 to 1000 μg/mL. Microscopic analysis showed that MPs internalization occurred after 24 h of exposure.

Another 2024 study suggests that the brain has a special capacity for microplastic accumulation compared to other organs. In the study by Campen et al. [456], Py-GC/MS was used to examine MPs from kidney, liver, and brain tissues. The brain had the highest concentrations of MPs, especially PE, with a wide range of sizes. While the liver and kidneys had 465 and 666 μg/g, respectively, the brain from the frontal cortex had concentrations of 3057 μg/g, 4806 μg/g, and even 8861 μg/g over the years studied. The mechanism of BBB crossing by MPs is not known but it has been proven to be possible, especially after BBB damage. Xie et al. [457] in a study of cerebrospinal fluid (CSF) samples collected from patients with and without CNS infection showed that PS, polyethylene PE, PP, and PVC penetrated the central nervous system (CNS), and PP and PE concentrations were positively correlated to the albumin index. The detection of MPs in the olfactory bulb indicates the possibility of bypassing the blood-brain barrier and translocation of MPs via the olfactory route. In the study by Amato-Lourenço et al. [458], the presence of MPs in brain tissue collected during the autopsy of 15 deceased persons was examined. The subject of the study was the olfactory bulb. After tissue digestion, the examination was performed using the µ-FTIR method. MPs in the form of particles and fibers were detected in 8 of 15 persons. The most abundant was one of the most commonly used polymers for clothing and packaging, PP, with sizes from 5.5 μm to 26.4 μm.

## 7. Conclusions and Future Perspectives

A consistent methodology should precede the assessment of MPs in human samples. Unfortunately, there are no standardized methods for the collection, preparation, quantification, and characterization of MPs. Therefore, results obtained using different test methodologies should not be compared, as the conclusion may be subject to significant error.

It has been shown that MPs can be absorbed and accumulate in distant tissues, leading to an inflammatory reaction, but the concentration of MPs causing health consequences in humans has not been determined. It is only known that MP levels in the environment are lower than the thresholds causing inflammation and stress in laboratory conditions.

In future studies on MP toxicity, in addition to the physicochemical properties of MPs, the so-called corona should be taken into account, which, due to its biocompatibility, facilitates the penetration and accumulation of MPs and the fact that MPs are a vector for many carcinogens.

The effect of MPs on the mechanisms of cancer development is the subject of in vitro studies on cell lines and in vivo studies using animal models. So far, changes in DNA repair mechanisms, cell proliferation, and cell death have been observed as a result of MPs exposure. One of the limitations of in vitro studies on the effects of MPs exposure is the common use of standardized PS beads, while in vivo studies are limited by the duration of the study, which usually does not exceed 3 months and refers to subchronic toxicity, while humans are continuously exposed to low levels of MPs, but throughout their lives. In addition, in vivo studies are mainly conducted on rodents, and there is a lack of studies involving mammals, which would be more reliable in anticipating the effects of exposure in humans.

## Figures and Tables

**Table 1:** Characteristics of plastic particles.

Particles Size	The Form	Chemical Identity	Additives	Contaminants
nanoparticles (≤100 nm), nanoplastics (100–1000 nm), microplastics (1 μm < 1000 μm), mesoplastics (0.5–5 cm), macroplastics (5–50 cm) megaplastics (>50 cm)	fragments, spheres, fibers, spheroids, granules, pellets, foam (polystyrene), flakes, and beads	polyurethane (PU/PUR), epoxy resins, vinyl esters, and silicones, polyvinyl chloride (PVC), polyethylene (PE), polypropylene (PP), polystyrene (PS), polyethylene terephthalate (PET), polyamide (PA), polycarbonate (PC), polyester (PES), polymethyl methacrylate (PMMA), ethylene-vinyl acetate (EVA), high-density polyethylene (HDPE), Low Density Polyethylene (LDPE), nylon (polyamide 6), polyethersulfon (PES), polyvinyl alcohol (PVA/PVOH), polysulfone (PSF/PSU), acrylonitrile-butadiene-styrene (ABS), polyacrylonitrile (PAN), cellulose acetate (CA), polytetrafluoroethylene (PTFE), polyoxymethylene (POM), styrene-ethylene-butylene-styrene (SEBS), expanded polystyrene (EPS), thermoplastic elastomere (TPE), polyfumaronitrile:styrene (FNS), chlorinated polyethylene (CPE)	fillers, plasticizers, heat and light stabilizers, antioxidants, coloring agents, lubricants, and flame retardants, antistatic agents, slip agents, biocides, and thermal stabilizers	adsorbing contaminants and pathogens from the environment

**Table 2:** Microplastic contamination of various categories of food products.

Kind of Products	Samples	MPs Content	Ref.
honey	19 honey samples from Germany, France, Italy, Spain, and Mexico	fibre: 166 ± 147 MPs/kg, fragments: 9 ± 9 MPs/kg	[76]
sugar	five commercial sugars	fibre: 217 ± 123 MPs/kg, fragments: 32 ± 7 MPs/kg	[76]
	sugar from Germany	249 ± 130 MPs/kg, <0.8 µm, PET, PE, PP	
fish	4 dried fish species	36 plastic polymers	[77]
	1337 specimens of fish	stomach or intestines: 1–35 MPs, 656 ± 803 μm	[78]
	*Siganus rivulatus*, *Diplodus sargus*, *Sardinella aurita*, *Sphyraena viridensis*, and *Atherina boyer*	28–7527 MPs/fish, ≤25–2000 µm, PVA, LDPE, HDPE, PET, PP, Nylon	[79]
salt	21 table salts from Spain	50–280 MPs/kg, PET, PP and PE	[80]
	salts from Taiwan	9.77 MP/kg, 1–1500 μm, PET, PP, PE	[81]
	sea salts from Bangladesh	2676 MPs/kg, 0.1–5 mm, PS, EVA, HDPE, Nylon, PET	[82]
water	ground water and drinking water	0.7 MPs/m^3^, 50–150 μm, PE, PA, PES, PVC, epoxy resin	[83]
	drinking water in plastic bottles, glass bottles, and cartons	PET, PP, 1–500 μm, 118 ± 88 MPs/L in returnable, 14 ± 14 MPs/L in plastic bottles, 11 ± 8 MPs/L in the beverage cartons, 50 ± 52 MPs/L in the glass bottles	[84]
	tap and bottled water	2649 ± 2857 MPs/L in PET bottles; 930 MPs/L tap water	[70,85,86,87,88]
seafood	bivalve mollusks	0.15 ± 0.20 MPs/g, 43–4720 µm, PE, PP, PS, PES	[89]
	mussel	0.040 ± 0.003 MPs/g wet weight (w.w.); 500 μm–2000 μm, PET, latex, PS-cotton, PVC, CA, EVA, HDPE, Nylon	[73]
	fish, oysters, and crustaceans	3.5 ± 0.8 MPs/fish 99.9 MPs/oysters 4.4 MPs crustaceans	[64,90,91,92,93,94,95,96,97,98,99,100,101,102,103,104]
	bivalves (*Mytilus edulis*, *Crassostrea gigas*)	*M. edulis*: 0.36 ± 0.07 MPs/g w.w. *C. gigas:* 0.47 ± 0.16 MPs/g w.w.	[64]
beverages	tea, soft drinks, energy drinks, beers	tea: PA, PEA, 100–2000 µm; 11 ± 5.26 MPs/L, soft drinks: PA, PEA, ABS, 100–3000 µm, 40 ± 24.53 MPs/L, energy drinks: PA, PEA, 100–3000 µm, 14 ± 5.79 MPs/L, beers: PA, PET, PEA, 100–3000 µm, 152 ± 50.97 MPs/L	[105]
	24 German beers	fibres: 2–79 MPs/L, fragments:12–109 MPs/L, granules: 2–66 MPs/L	[106]
milk	23 milk samples from Mexico	6.5 ± 2.3 MPs/L, 0.1–5 mm, PES, PSU	[107]
fruits, and vegetables	pear, apple, lettuce, broccoli, carrot	fruits: 52,600–307,750 MPs/kg, 1.81–2.29 μm vegetables: 72,175–130,500 MPs/kg, 1.51–2.52 μm	[108]
meat	chicken	4–18.7 MPs/kg, 130–450 μm, extruded polystyrene (XPS)	[109]
rice	rice from a supermarket in southeast Queensland, Australia	dry rice: 67 ± 26 μg/g dry weight (d.w.) washed rice: 52 ± 5 μg/g d.w. dry instant rice: 280 ± 50 μg/g d.w. washed instant rice: 170 ± 41 μg/g d.w. PE, PP, PET	[110]

**Table 3:** Sample treatment and analysis techniques used for detection of MPs in human matrices.

Biological Matrix	Sample Digestion Steps	Identification Method	MPs Size	Ref.
Urinary system				
urine	10% KOH at a ratio of 1:2 (sample/KOH, *v*/*v*) for 48 h at 40 °C, filtration through a glass fiber filter	binocular microscopy, µRaman	4–15 µm	[165]
urine	30% H_2_O_2_ and 50 mM acidified iron (II) sulfate heptahydrate (Fenton’s reagent) at a ratio of 1:2.5 and 6 g of NaCl heated at 50 °C for 30 min; after cooling to room temperature for 24 h, the sample was filtrated by a 0.45 µm pore-size PTFE filter paper	optical microscopy, FTIR, µRaman, SEM-EDS	0.01–0.34 nm (fragments), 10–871 µm (the lengths of the fibers) 1.59 ± 1.80 fragments/100 mL, 2.04 ± 3.38 microfibers/100 mL	[166]
kidney, (spleen, liver)	10 M KOH and sodium 6–14% hypochlorite in a 2:1 ratio was added (5 mL/g w.w.) at 40 °C for 72 h (two times); then defatting with acetone, and filtration via silver membrane filters	fluorescent microscopy, µRaman	10–20 μm, spleen (0.9 MPs/g), liver (3.2 MPs/g), kidney (0.2 MPs/g)	[167]
Cardiovascular system				
saphene vein tissue	30% H_2_O_2_ was shaken for 168 h at 65 °C, at 85 rpm; then filtration with Al_2_O_3_ filters of 0.02 µm	μFTIR—transmittance mode	16–1074 μm 14.99 ± 17.18 MPs/g	[168]
heart tissues, and blood before and after cardiac surgery	30% H_2_O_2_ was added to sample (1:5, *v*/*v*), shaken at 120 rpm for 12 h/day for 20 days, digested with 68 wt% HNO_3_ and 10 wt% NaOH while homogenizing in an ultrasonic bath at 40 kHz for 30 min, rinsed with water, and filtered through a 1 μm PTFE membrane	LIDR	20–469 μm (tissues) blood (184 μm)	[169]
blood vessels	1 mL of whole blood with 15 mL of 400 mM TRIS-HCl buffer heated at 60 °C for 1 h, then 100 µL of the Proteinase K (1 mg/mL, 3.0–15.0 unit/mg), and 1 mL of 5 mM CaCl_2_, for 2 h, then shaken for 20 min. at room temperature and heated at 60 °C for 20 min, filtration by a glass fiber filter (diameter 25 mm, mesh size 700 nm) and rinsing with 30% H_2_O_2_ solution	Py-GC/MS	>700 nm, 1.6 µg/mL	[170]
thrombi	30% KOH solution at 60 °C for 4 h and room temperature for 48 h, then filtered with the 0.7 μm-pore glass filter	Raman	2.1–26.0 μm, 1–15 MPs detected in thrombus	[171]
Reproductive system				
testis, semen	10% KOH solution for 48 h at 40 °C, then the filtration under vacuum by 1.2 μm pore-size glass filter membranes	Py-GC/MS and LDIR	21.76 μm to 286.71 μm, 0.23 ± 0.45 particles/mL in semen and 11.60 ± 15.52 particles/g in testis	[172]
semen	10% KOH solution in a 1:2 ratio (*v*/*v*, sample/KOH) for 48 h at 40 °C, then the filtration under vacuum by 1.2 μm pore-size glass microfiber filter membranes	µRaman	2 to 6 μm, 16 MPs fragments were found in six of ten samples	[173]
placenta	10% KOH in a ratio of 1:30 (*w*/*v*) for 72 h at 50 °C and 120 rpm, filtration to 10 µm stain steel sieve, and rinse with water and ethanol	LDIR imaging–Reflection	20–500 µm, 2.70 ± 2.65 particles/g	[174]
placenta	10% KOH to the sample in a ratio of 1:8 (*w*/*v*), then incubated at room temperature for 7 days, then filtered through a 1.6 µm-pore-size filter membrane	µRaman	5–10 μm, 12 fragments were found in 4 placentas	[175]
breast milk	10% KOH in a ratio of 1:10 (*w*/*v*) at 40 °C for 48 h, then filtered through a 1.6 µm pore-size filter membrane	µRaman	2–12 µm, MPs found in 26 out of 34 samples	[176]
breast milk	10% KOH solution was added to the sample and incubated at 40 °C for 48 h, then vacuum filtered through a 1.6 μm membrane.	µRaman	MPs were detected in 23 of 59 of samples.	[153]
Respiratory system				
pulmonary tissue	digestion with enzymatic mixture Corolase^®^ 7089 (20 UHb/mL for sample) for 12 h at 60 °C, density separation by ZnCl_2_ solution (1.5 g cm^−3^), filtration by silver membrane	Binocular microscopy, Raman	polymeric particles < 5.5 µm (n = 33) and fibres 8.12 to 16.8 µm (n = 4) were observed in 13 of 20 tissue samples.	[177]
lung tissue	digestion with 100 mL of 30% H_2_O_2_ and shaking at 55 °C for 11 days at 65 rpm; after 5 days, 100 mL of 30% H_2_O_2_ was added then filtered onto aluminum oxide filters	μFTIR	12–2475 μm, 0.69 ± 0.84 MP/g	[178]
lung granule nodules	30 mL of 30% H_2_O_2_, and shaken for 72 h incubation with 80 rpm at 65 °C, then filtered through a 5 μm filter membrane	μFTIR, Raman	>20 μm	[179]
BALF	drying at 60 °C overnight on glass Petri dishes with no digestion procedure	µFTIR, SEM-EDS, Microscope	1.73 ± 0.15 mm, with the longest dimension (9.96 mm)	[180]
Lymphatic system				
spleen	10 M KOH and NaClO in a 2:1 ratio were added and heated at 40 °C for 72 h, then filtered via silver membrane filters	fluorescence microscopy, µRaman	5–25 μm	[167]
Digestive system				
colorectal cancer tissue	digestion with 10% KOH at 60 °C for 7–10 h, then diluted with deionized water and filtered using 0.45 μm cellulose membrane paper	Stereomicroscope (hot needle test) µFTIR -ATR mode SEM–EDX	0.8–1.6 mm, 28.1 ± 15.4 MPs/g	[181]
liver tissues	digestion with 10% KOH and NaClO 2:1 ratio at 40 °C for 72 h, filtration through silver filter, second digestion with 30% H_2_O_2_, filtration through silver filter	Fluorescent microscopy and Red Nile staining µRaman	4–30 μm	[167]
feces	samples dried to constant weight (1 g) at 70 °C for at least 1 week and 50 mL of Fenton’s reagent at room temperature was added for 5 h, filtration using a cellulose nitrate-cellulose acetate filter with a pore size of 0.8 μm, filter digestion with 65% HNO_3_ at 50 °C for 30 min, incubation at 70 °C for 10 min, dilution with distilled water in a ratio of 1:2	Raman	<5 mm, 10.19 μg/g	[182,183]
feces	lyophilized samples and Fenton’s reagent (30% H_2_O_2_ and iron catalyst solution (20 g iron(II) sulfate heptahydrate in 1 L water) were mixed in a volume ratio of 1:2.5 and left for 5 h below 40 °C, filtered through CN-CA filters (diameter 47 mm, pore size 1 μm), the filter incubated with 65% HNO_3_ at 50 °C for 30 min, and then diluted with water in a ratio of 1:2 (*v*/*v*) and filtered through PTFE membrane (diameter 47 mm, pore size 1 μm)	μRaman	1.7–393.8 μm, 41.8 MPs/g dm) (IBD); 4.4–333.2 μm, 28.0 MPs/g dm (healthy adult)	[184]
feces	samples 3.0 g ± 0.1 g left with 25 mL of 30% H_2_O_2_ for 20 days, freeze drying, sieving of particles > 5 mm, filtration through a polycarbonate microporous membrane	µFTIR Reflection mode	20 to 800 μm, from 1 MPs/g to 36 MPs/g	[185]
feces	samples 0.32 ± 0.14 g, 450 mL of 10% KOH and 15% EDTA solution left at 40 °C for 24 h, then incubated with 50 mL of 30% H_2_O_2_ for 48 h; digestion of filtered cellulose fibers with 50 mL of 2% AMIM-Cl for 24 h; filtration and flotation in 50 mL of sodium iodide solution (3.67 g cm^−3^), filtration through a stainless steel sieve with pores of 30 µm	Raman	40.2–4812.9 μm, 20.4–138.9 MPs/g w.w.	[186]
placentas and meconium	68% HNO_3_ was added and left for 48 h, then heated for 3 h at 95 °C, filtered by vacuum through 13 μm stainless steel membranes, then the membrane was rinsed with ultrapure water and anhydrous ethanol, then the membrane was sonicated in anhydrous ethanol for 30 min, the filtrate was filtered and concentrated to 200 μL	LIDR	20–50 μm, in the placenta was 18.0 MPs/g, In the meconium was 54.1 MPs/g	[187,188]
Integumentary system				
saliva	35 mL of 35% H_2_O_2_ was added for 2–10 days with heating, density separation with 50 mL of ZnCl_2_ (1.6 g/cm^3^), shaking for 5 min at 350 rpm, sedimented for 90 min, centrifuged for 3 min at 4000 rpm, and then vacuum filtered through 2 μm blue filter papers, centrifugation, and vacuum filtration	Binocular microscopy, Polarized light microscopy Fluorescence microscopy, µRaman	fibres of 100–500 μm in length, 0.33 MPs per individual	[189]
sputum	digestion with HNO_3_, density separation with ZnCl_2_ (1.7–1.8 g/cm^3^), filtration in a silver membrane, rinsing and soaking in ethanol, drying	Optical microscope, µFTIR LDIR	20–500 µm (LDIR), >0.1 mm (µFTIR), 18.75–91.75 MPs/10 mL	[190]

Abbreviations: inflammatory bowel disease (IBD); wet weight (w.w.); polytetrafluoroethylene (PTFE); Fourier-transform infrared spectroscopy (FTIR); µRaman, scanning electron microscope and energy-dispersive X-ray spectroscopy (SEM-EDS); Raman micro-spectroscopy (μRaman); the micro Fourier Transform Interferometer (μFTIR); pyrolysis-gas chroma-tog-raphy/mass spectrometry (Py-GC/MS), laser direct infrared spectroscopy (LDIR); bronchoalveolar lavage fluid (BALF); cellulose nitrate-cellulose acetate (CN-CA); inflammatory bowel disease (IBD); 1-allyl-3-methylimidazolium chloride (AMIM-Cl).

**Table 4:** The imaging methods that enable the rapid identification of MPs.

Technique	Particle Size	Advantages	Limitations
SEM with energy dispersive X-ray spectroscopy (SEM-EDX)	<1 µm	surface characteristics and chemical composition of MPs, the number of MPs, identification of inorganic additives	cannot be used to calculate the mass of MPs in the sample, destructive to the MPs, time-consuming
µRaman	>1 µm <20 μm	characterize MPs morphology as well as their chemical composition, non-destructive to the MPs.	small amount of sample, requires extensive sample pre-treatment.
µFTIR	>5 µm <500 μm	the ability to perform IR spectroscopic analysis on discrete microscopic regions of a sample, the ability to map the distribution of different chemical species in heterogeneous samples	limited in spatial resolution, a large incoherent source cannot be focused onto a small microparticle, weak signals, slow analysis, it struggles to detect plastics below 20 μm, yields inaccurate spectra for highly weathered plastics, influence of other materials adhered on the microplastic particles, ATR-FTIR can destroy the sample
O-PTIR	>~500 nm (0.42 µm, theoretical detection limit)	a theoretical spatial resolution of ~416 nm is independent of IR wavelength; sub-micron IR spectroscopy is possible with co-located Raman and Fluorescence, the spectral range of ~1800 cm^−1^ to ~800 cm^−1^ could be extended by changing or adding an additional IR source; high SNR; immune to spectral artifacts.	the identification of O-PTIR using less sampling area and single wavenumber might result in the exaggeration of the MPs numbers and incorrect identification of the particles, slow imaging speed.
the QCL-IR microscope	>10.6 (10.9) µm	a large field of view, the scanning speed of QCL-IR is faster than O-PTIR, high SNR, very fast imaging speed (SPERO microscope), high automation, and integration	lower resolution in comparison to O-PTIR, narrow spectral range of ~1800 cm^−1^ to ~800 cm^−1^, susceptible to spectral artifacts, slow imaging speed (LDIR), extensive sample pre-treatment.

**Table 5:** In vitro and in vivo studies on direct/indirect effect of NMPs on carcinogenesis.

Model	NMPs	Observations	Ref.
in vitro (human cell lines)			
differentiated human colorectal adenocarcinoma cells, Caco-2	PS-MPs (20 and 40 nm) with two different surface chemistries (carboxylic acid and amines)	losses in cell viability, apoptosis induction	[300]
human breast cancer cell lines (MCF-7, MDA-MB-231)	PP pellets, <100 µm, at concentrations of 1.6 mg/mL for 24 h	enhance metastasis-related gene expression and cytokines, promotion of metastatic features	[301]
gastric adenocarcinoma (AGS) cells	PS-MPs (44 nm and 100 nm) at concentration of 10 µg/mL (1 h)	changes in cell viability, inflammatory gene expression, and cell morphology, 44 nm MPs strongly induced upregulation of IL-6 and IL-8 genes	[302]
gastric cancer cell lines (AGS, MKN1, MKN45, NCI-N87 and KATOIII)	PS-MPs (9.5–11.5 µm)	increased proliferation, invasion, and migration of cancer cells resistant to anticancer drugs (borte-zomib, cisplatin, paclitaxel, gefitinib, lapatinib, and trastuzumab) due to increased expression of asialoglycoprotein receptor 2 (ASGR2) in mice with transplanted PS cells (NCI-N87)	[303]
the human colon adenocarcinoma Caco-2 cells	PS-MPs 0.1 µm (≥20 µg/mL) and 5 µm (≥80 µg/mL)	low toxicity on cell viability, oxidative stress, and membrane integrity and fluidity, disruption of the mitochondrial membrane potential (5 µm PS-MPs > 0.1 µm PS-MPs); the 0.1 μm PS-MPs act as substrates of ABC transporter, and 5 μm PS-MPs might reduce ABC transporter activity	[304]
Calu-3 cells (human lung epithelial cells) and THP-1 cells (human macrophage cells)	50 nm aminated PS nanospheres	DNA damage	[305]
a human colon cell line (CCD-18Co)	PS-MPs (0.5 and 2 µm)	the metabolic changes regarding glucose and glutamine metabolism and induction of oxidative stress similar to the effect of azoxymethane (AOM) in a human colon cancer cell line (HCT15)	[306]
the human fibroblast Hs27 cell line	PS-MPs (100 nm) with a concentration of 5, 25, and 75 μg/mL (15, 30, 45, 60 min, and 24, 48 h)	the DNA damage, increased levels of MN and nuclear buds, and an increase in ROS production are responsible for the genotoxic effect	[288]
Caco-2 monolayer cells	PS-MPs (100 nm and 5 μm) at concentrations of 1 and 10 µg/mL	cytotoxicity, disorders of transport function, and increased proinflammatory	[307]
the triple culture model (Caco-2/HT29-MTX-E12/THP-1)	PS-MPs (50 nm, 100 nm) and PVC-MPs (<50 μm)	an increase in the release of IL-1β and a loss of epithelial cells	[308]
human-derived HDFs, HMC-1 cells, PBMCs, and other cells	PS-MPs (10–100 µm) at concentrations between 0.5 and 1000 µg/mL	no significant cytotoxicity, induction of pro-inflammatory cytokines by smaller PS-MPs	[289]
HepG2CDKN1A-DsRed biosensor cells CHO-k1 (placental barrier model)	50 nm and 0.5 μm COOH-modified PS-MPs; 0.01, 0.1, 1, 5, 10, 25, and 50 μg/mL (24 and 48 h)	lack of transport across the intestinal and placental barriers, intracellular accumulation of MPs, weak embryotoxicity, no genotoxicity, lack of p53 expression, and MN induction	[309]
Caco-2 cells	PS-MPs (50 nm and 200 nm) at concentrations between 15 and 250 μg/mL for up to 120 min of exposure	no cytotoxicity	[310]
Caco-2 cells	PET-MPs sized 100 nm at 1–30 µg/mL concentrations after 24 h of incubation	no cytotoxicity	[311]
Caco-2 cells	PS-MPs with a concentration of 12.5 mg L^−1^ or 50.0 mg L^−1^ for 24 h	decrease cell viability in a dose-dependent manner	[295]
Caco-2 cells and Caco-2-based cell-cultures	PS-MPs with the sizes of 1 and 10 µm	cytotoxicity only in concentrations 1 × 10^8^ MPs/mL	[261]
Caco-2/HT29-MTX-E12 with human macrophages and dendritic cells	MPs (PP, PU, PA, and tire rubber polydisperse) (50–500 µm) up to 48 h exposure	any significant cytotoxicity or releasing inflammatory cytokines	[312]
human leukocytic cell lines: Raji-B (B-lymphocytes), TK6 (lymphoblasts), and THP-1 (monocytes)	spherical PS-MPs of about 50 nm, 5, 10, 25, and 50 μg/mL (3, 24, 48 h)	the monocytic THP-1 cells exhibited the highest PS-MPs internalization, and no adverse effects, Raji-B and TK6 dispense lesser uptake, showed mild toxicity, ROS production, and genotoxicity	[313]
skin squamous cell carcinoma cell lines (SCL-1 and A431), HaCaT cells in a normal skin cell model	PE-MPs (1 µm) at concentrations of 0–1 mg/mL	time- and dose-dependent internalization of MPs, induction of proliferation by NLRP3 activation, increase in mitochondrial ROS, change of mitochondrial membrane potential in skin cancer cells, damage to normal skin cells by NLRP3-induced inflammation, and burn death.	[314]
human colorectal cancer cell lines (HT29, HCT116, SW480, and SW620)	PS-MPs (0.25, 1, and 10 μm) at concentrations of 0.16–5 μg/mL for 72 h	a significant size- and concentration-dependent MPs uptake, the highest uptake for HCT116, and no signs of MPs elimination from the cells. Particles were distributed between mother and daughter cells during cell division, amplified cell migration after short-term exposure to 0.25 μm MPs, and prometastatic effects	[315]
the human EOC cell line HEY	PS-MPs (100 nm; 10 mg/L)	reduction of the relative viability of EOC cells in a dose-dependent manner	[316]
Human lymphocytes	PS-MPs, plasma coronated-PS-MPs, scrub isolated-PS-MPs 100 nm, 1, 2.5, 5, 7.5 and 10 μg/mL (24 h)	DNA damage induction	[317]
human colonic epithelial cell CCD841CoN and small intestinal epithelial cell HIEC-6	0.1, 0.5, 1, 5 μm microspheres PS-MPs and nanospheres within 24 h	nanospheres entered cells more than PS-MPs; PS-MPs damaged the membrane and caused mitochondrial depolarization more than that of nanospheres, with low toxicity to CCD841CoN and HIEC-6 cells.	[318]
the cultured human alveolar A549 cells	(PS-MPs) of 1 and 10 μm at concentrations of 0.05-100 μg/mL for 24, 48, 72, and 96 h of exposure	significant reduction in cell proliferation, little cytotoxicity, the high viabilities, decrease in metabolic activity, major changes in the morphology of cells after 24 h, the uptake of 1 μm PS-MPs into the cells.	[319]
GES-1 cells	chlorinated PS-MPs to those of pristine PS-MPs	chlorinated PS-MPs inhibited the cell proliferation, changed cellular morphology, destroyed cell membrane integrity, induced cell inflammatory response and apoptosis by the regulation of PI3K/AKT and Bcl-2/Bax pathways, oxidative stress-triggered mitochondrial depolarization, and the activation of caspase cascade	[320]
intestinal epithelial Caco-2, lung epithelial A549, the innate (THP-1, U937 macrophage), and adaptive (Jurkat T cell line)	(30.5 ± 10.5 and 6.2 ± 2.0 μm) PE-MPs at concentration of 1–1000 μg/mL	reduction of cell viability in intestinal epithelial Caco-2 and lung epithelial A549 cells by 1000 μg/mL MPs, induction of NO and ROS (THP-1, Jurkat, U937), cytokine response in HaCaT	[121]
human blood lymphocytes	PVC-MPs at 24, 48, and 96 μg/mL for 3 h	ROS formation, lysosomal membrane injury, mitochondrial MMP collapse, depletion of glutathione, and lipid peroxidation.	[321]
human peripheral lymphocyte cells	PE- MPs (10–45 μm), at concentrations of 25, 50, 100, 250, and 500 μg/mL for 48 h of exposure	increase in the level of genomic instability with a lack of cytotoxic potential, and increase in MN, NPB, and NBUD frequencies	[322]
Caco-2	PS-MPs 50 nm, 0.26, and 6.5 μg/cm^2^ (24 h) and 0.0006, 0.26, 1.3, and 6.5 μg/cm^2^ (8 weeks)	lack of DNA damage induction and oxidative stress induction	[323]
Caco-2/HT29, Caco-2/HT29/Raji-B	PS-MPs (50 nm) at concentrations of 1, 25, 50, and 100 μg/mL (24 h)	lack of DNA damage induction and oxidative stress induction	[324]
PBMCs, HMCs-1, RBCs, HDFs, HeLa cells	PVC, ABS from 25 to 75 μm and from 75 to 200 μm; at concentrations of 5–1000 μg/mL from 1 to 5 days	IL-6 and TNF-α release at all concentrations	[325]
RBCs, PBMCs, Raw 264.7 mouse macrophage cell line, HMC-1 cell lines	PP-MPs of ~20 μm at concentrations of 2 mg/mL and 25–200 μm at concentrations of 0.1, 0.3, 1.5, 3.0, and 4.5 mg/well and cultured for 48 h	increase in cytokine and histamine levels, cytokines (IL-6, TNF alpha, and histamine)	[294]
human hepatoma cell line HepG2	dioxin-like PCB congeners (PCB 101, PCB 126) at concentrations of PCB 126: from 0.6 to 25 µM; PCB 101: from 1.2 to 50 µM, and 48 h of exposure	single PCB exposures cause changes in glycerophospholipids and glycerolipids in a dose-dependent manner; MPs cause an increase in triglyceride content; and combined exposures cause more harmful effects	[326]
human forebrain cortical spheroids (Undifferentiated human iPSC culture)	PS-MPs exposed to 100, 50, and 5 µg/mL of 1 µm and 10 µm during day 4–10 and day 4–30.	promotion of proliferation and high gene expression (Nestin, PAX6, ATF4, HOXB4, and SOD2) after the short-term MP exposure, reduction of cell viability, decrease in β-tubulin III, Nestin, and TBR1/TBR2 gene expression after long-term exposure	[327]
human alveolar epithelial A549 cell line	PS-MPs: 25 nm (25 µg/mL) and 70 nm (160 µg/mL) within 8 h	Inflammatory response: increased expression of IL-6, IL-8, NF-κβ, and TNF-α; Proliferation: Increased expression of CCND (cyclin D), CCNE (cyclin E), and Ki67	[328]
liver organoids	PS-MPs 1 μm microbeads 0.25, 2.5, and 25 μg/mL within 48 h	Inflammatory response: increased expression of IL-5, increased expression of COL1A	[329]
colonic cancer Caco-2 cells	300 nm, 500 nm, 1 μm, 3 μm, 6 μm PS-MPs	increased cellular oxidative stress and mitochondrial depolarization; MPs caused an increase in ROS and synergistic toxicity with bisphenol A; MPs (1 µm, 3 µm) decreased MPs, lower toxicity, and higher uptake rate	[330]
human intestinal cell lines (Caco-2 and NCM 460)	0.1 and 1 µm PS MPs for 24 h exposure	internalization of both PS MPs, no changes in cell viability, ROS levels and nutrient uptake/metabolism, alteration of redox homeostasis (NCM 460)	[331]
Lymphocytes, monocytes, PMNCs	PS-MPs 50 nm, 50, and 100 μg/mL (24 h)	lack of lymphocytes DNA damage induction, monocytes and PMNCs DNA damage	[290]
Human skin explants obtained from elective abdominalplasties	500 µg/mL 20 nm PS-MPs was first applied to skin biopsies using a glass rod. Skin biopsies were incubated for 24 h	disorders of skin redox homeostasis, modulation of inflammasome pathways (NLRP1, NLRP6)	[332]
vaginal keratinocytes	PE-MPs (200 nm to 9 μm) at 25 and 250 μg/mL	altered expression of junction and adhesion proteins, actin cortex organization, levels of genes involved in oxidative stress signaling pathways and miRNAs related to epithelial barrier function, altered expression of DNA methyltransferase and DNA demethylase	[333]
in vivo			
mice	oral exposure to PS-MPs (0.5 and 50 μm) at concentrations of 100 and 1000 μg/L for 35 days	decrease in mucin secretion in the gut, induction of gut microbiota dysbiosis, and hepatic lipid metabolism disorder	[334]
mice	PS-MPs (10–50 nm), 300 μg/mouse (intranasal), every 3 days for 24 days	increased expression of IgG1 and TNF-α, eosinophils and lymphocytes infiltration	[335]
mice	PS-MPs (500 nm), 5, 25, and 50 µg/ mouse (p.o.) daily for 2 weeks	upregulation of the ASC inflammasome and NF-κβ pathways, increased expression of NF-κβ (25 and 50 µg), IL6, TNF-α, IL-1β, TGF-β, and IL-10	[336]
mice	PE (10–150 µm), 6, 60, and 600 µg/ mouse (p.o.), daily for 5 weeks	increased expression of IL-2 and IL-6 (6 µg), IP-10 and RANTES (60 µg), IL-5 and IL-9 (600 µg), G-CSF (60 and 600 µg), and IL1α; TLR4, AP-1, IRF5 (600 µg), and lymphocytes and plasma cells infiltration (600 µg); decrease percentage of Th17 and Treg cells (60 and 600 µg)	[337]
mice	PS-MPs (10 µm), 250 µg/mouse (i.p.), twice on 5.5 and 7.5 days of gestation	increased activity of AST and ALT (high-fat diet-fed mice, 1 μg), increased macrophage infiltration and increased collagen deposition (high-fat diet fed and normal mice, 1 µg), increased expression of IL-1β (high-fat diet mice, 1 and 5 μg), IL-12, IL-2, and IFN-γ (high-fat diet fed mice, 1 μg), increased expression of α-SMA and Col1a (high-fat-diet fed mice, 1 μg)	[338]
mice	PS-MPs ( 42 nm), 1 and 5 μg/mouse (i.v.), every 3 days for 15 days	increased activity of AST and ALT (high-fat diet-fed mice, 1 μg), increased macrophage infiltration and increased collagen deposition (high-fat diet-fed and normal mice, 1 µg), increased expression of IL-1β (high-fat diet mice, 1 and 5 μg), IL-12, IL-2, and IFN-γ (high-fat diet fed mice, 1 μg); increased expression of α-SMA and Col1a (high-fat diet-fed mice, 1 μg)	[339]
rats	PS-MPs (500 nm), 0.5, 5, and 50 mg/L of drinking water/rat (p.o.), daily in drinking water for 90 days	increased expression of Wnt, TGF-β, p-β-catenin, α-SMA, Collagen I and fibronectin (5 and 50 mg/L) and β-catenin and Collagen III (50 mg/L); increased collagen (50 mg/L) and fibronectin (5 and 50 mg/L) deposition	[340]
mice	PS-MPs (5, 20 μm), 0.01, 0.1, and 0.5 mg/day/mouse (p.o.), Daily for 1, 2, 4, 7, 14, 21, and 28 days	inflammation indicators observed (0.5 mg/d, 28 days)	[341]
mice	PLGA (1–2 μm), 100 μg/mouse (s.q.), once a week for 5 weeks	increased expression of IL-10 and TGF-β1, INF-γ and IL-17A, augmentation of Treg and TGF-β1 release	[342]
mice	PS- MPs (5 μm) at 100–1000 mg/L for 6 weeks	an increase in bile acid secretion in the liver and a decrease in mucus secretion in the colon	[343]
mice	PS (5 μm) at a concentration of 500 μg/L for 28 days	an increase in intestinal permeability with acute colitis and lipid disorders in the liver	[344]
mice	PS-MPs (0.5 μm) at a concentration of 0.6–0.7 μg/day, 6–7 μg/day, and 60–70 μg/day for 35 days	testicular toxicity and spermatogenesis disorders by inducing inflammation after exposure	[345]
mice	PS-MPs (0.5, 4, and 10 μm) at a 10 mg/mL concentration for 28 days	induced testicular inflammation, the blood-testis barrier disruption, and decreased testosterone levels	[346]
mice	PS-MPs (5.0–5.9 μm) at doses of 0.01, and 0.1, 1 mg/d for 40 days	reproductive toxicity through oxidative stress in testicles and sperm damage	[347]
mice	PE (0.4–5 µm) at a concentration of 100 mg/kg for 30 days) contaminated phthalates	reproductive toxicities by the testicular transcriptomic alterations	[260]
mice	PS-MPs, 9.5–11.5 µm; 1.72 × 10^4^ particles/mL/mouse (oral, once), 8.61 × 10^5^ particles/mL 4-weeks-exposed NCI-N87 (subcutaneously-4 weeks	accelerated tumor growth (4 weeks); altered expression of 194 genes associated with digestive system diseases and cancer (once)	[303]
the offspring of mice during the gestation and lactation periods	PS-MPs (0.5 and 5 μm) at concentrations of 100–1000 mg/L for 6 weeks	increases the risk of metabolic disorders, gut microbiota dysbiosis, and barrier dysfunction	[348]
mice	PE-MPs (10–150 μm) at concentrations of 6, 60, and 600 μg/day for 5 weeks	induces minor intestinal inflammation and increases the secretion of IL-1α in the serum	[337]
mice	ZnO-NPs and PS-MPs), 14.6 ng/kg b.w. for 3 days	increases nitric oxide levels, TBARS, reduction in acetylcholinesterase activity, and the accumulation of NPs in the brains; erythrocyte DNA damage; hypercholesterolemic and hypertriglyceridemic conditions	[349]
rats	PS-MPs (0.5 μm) at doses of 0.015, 0.15, and 1.5 mg/day after 90 days	granulosa cell apoptosis and fibrosis in the ovaries through oxidative stress, inflammatory dose-dependent response: increased expression of Wnt and TGF-β, β-catenin, p-β-catenin, α-SMA, Collagen I, fibronectin, and Collagen III	[350]
rats	PS-MPs 38.92 nm at doses of 1, 3, 6, and 10 mg/kg for 35 days	reproductive toxicity and a significant down-regulation of PLZF, DAZL, FSH, and LH gene expressions, as well as endocrine disturbances and histological lesions	[351]
mice	PE-MPs (0.5–1.0 μm) and OPFRs after 90 days exposure of compounds (MP: 2 mg/L, OPFRs + PS: 10 μg/L and 100 μg/L)	oxidative stress, neurotoxicity, and enhanced disruption of amino acids as well as energy metabolism	[352]
rats	PS-MPs (0.5 μm) at concentrations of 0.5, 5, and 50 mg/L for 90 days	cardiovascular toxicity by inducing cardiac fibrosis and myocardium apoptosis	[353]
zebrafish (Danio renio)	PE and PS-MPs (25–90 μm) for 20 days	alterations in immune system, lipid metabolism, and behavior	[354]
zebrafish (Danio renio)	PS-MPs (70 nm, 5 μm, and 20 μm) exposure for 7 days	oxidative stress through the release of reactive oxygen species and disorders of metabolic profile in the liver with alterations in lipid and energy metabolism	[265]
zebrafish (Danio renio)	PS-MPs at concentrations of 4 × 10^4^ and 4 × 10^6^ MPs/m^3^ for 5 days	gastrointestinal toxicity, oxidative stress, and behavior disorders	[355]
zebrafish (Danio renio)	MPs (1 μm) at concentrations of 10, 100, and 1000 μg/L after 21-day	changes in steroidogenic mRNA expression in gonads and the cumulative number of eggs spawned as well as fertilization rate, insignificant or recoverable transgenerational effects on offspring survival and early development	[356]
mice	PS-MPs (100 nm; 10 mg/L)	acceleration of epithelial ovarian cancer (EOC) tumor growth, increase mitotic counts in EOC tumor tissues, disturbance of the expression of thrombomodulin (THBD)	[316]
the bivalve *Mytilus galloprovincialis*	PE- and PS-MPs, <100 μm MPs with pyrene (50 μg/L) in rotating conditions for 6 days	acumulation of MPs in haemolymph, gills and digestive tissues, decrease in the expression of transcription genes related to apoptosis, cellular effects concering immunological responses, lysosomal compartment, peroxisomal proliferation, antioxidant system, neurotoxic effects, onset of genotoxicity	[292]
rats	PS-MPs microspheres with particle sizes of 80, 200, 500, and 1000 nm after (0 h, 6 h, 12 h, and 24 h) exposure	accumulation of MPs in gastric tissues, damage to gastric barrier and mitochondria, decrease in antioxidant enzyme activity, increase in MDA, 8-OhdG, and γ-H2AX, upregulation of β-catenin/YAP	[293]
juvenile Nile tilapia (*Oreochromis niloticus*)	1 mg/L, 10 mg/L, and 100 mg/L for 15 days	alterations in the activity of superoxide dismutase, catalase, total peroxides, total antioxidant capacity, lipid peroxidation, DNA fragmentation, and the electrophoretic pattern of muscle proteins	[291]
zebrafish larvae	fluorescent carboxylate PS-MPs of 50 nm and 100 nm at concentration of 0, 200, 400, 600, 800 i 1000 ppm for a period of 96 h	50 nm PS-MPs accumulated in the brain, intestine, and blood vessels, whereas 100 nm PS-NPs did not. 627.27 (±14.01) ppm of MPs was detected in the larvae exposed to 50 nm PS-NPs at 1000 ppm, whereas 160.25 (±36.82) ppm was detected in the larvae exposed to 100 nm PS-MPs	[357]
zebrafish	PS-MPs of ~70 nm at concentration of 0.5 ppm, 1.5 ppm, and 5 ppm, acute (~7 days) and chronic (~30 days and ~7 weeks) exposure	PS-MPs accumulated in the gonads, intestine, liver, and brain. PS-MPs caused disturbance of lipid, energy metabolism, and oxidative stress. Neurotransmitter expression in the brain was inhibited (Ach, DA, melatonin, GABA, 5-HT, vasopressin, kisspeptin, and oxytocin)	[358]
mice	PS-MPs (0.4–0.6 μm), at a concentration of 100 μg/mL, 0.5 mL/day, three times a week for 9 weeks	alterations in the lipid accumulation, adipogenesis, lipogenesis, and lipolysis pathways in the liver tissue of MP-treated mice; an upregulation of the serum lipid profile; an increase in leptin in the adipose tissues; disruptions in the glycogenolysis; the Glu transporter type 4 (GLUT4)-5′ AMP-activated protein kinase (AMPK) signaling pathway; levels of lipid intermediates; and the insulin resistance of the liver tissues	[359]

Abbreviations: micronucleation (MN), nucleoplasmic bridge formation (NPB), and nuclear bud formation (NBUD), acrylonitrile butadiene styrene (ABS), peripheral blood mononuclear cells (PBMCs), human mast cells (HMCs-1), and red blood cells (RBCs), normal cells (HDFs), and cancer cells (HeLa cells), interleukin 6 (IL-6), tumor necrosis factor-α (TNF-α), polypropylene (PP), plasma membrane ATP-binding cassette (ABC) transporter, thiobarbituric acid reactive species (TBARS), polyethylene (PE), mitochondrial membrane potential (MMP), intravenous (i.v.), organophosphorus flame retard-ants (OPFRs), body weight (b.w.), subcutaneous (s.q.), per os, by mouth or orally (p.o.), intraperitoneal (i.p.), zinc oxide nano-particles (ZnO-NPs).

## Data Availability

No new data were created.
